# Development of a predation index to assess trophic stability in the Gulf of Alaska

**DOI:** 10.1002/eap.2141

**Published:** 2020-07-31

**Authors:** Cheryl L. Barnes, Anne H. Beaudreau, Martin W. Dorn, Kirstin K. Holsman, Franz J. Mueter

**Affiliations:** ^1^ College of Fisheries and Ocean Sciences University of Alaska Fairbanks 17101 Point Lena Loop Road Juneau Alaska 99801 USA; ^2^ Status of Stocks and Multispecies Assessment Program Alaska Fisheries Science Center National Marine Fisheries Service National Oceanic and Atmospheric Administration 7600 Sand Point Way NE, Bldg 4 Seattle Washington 98115 USA; ^3^ Resource Ecology and Ecosystem Modeling Program Alaska Fisheries Science Center National Marine Fisheries Service National Oceanic and Atmospheric Administration 7600 Sand Point Way NE, Bldg 4 Seattle Washington 98115 USA

**Keywords:** ecosystem‐based fisheries management, food web ecology, groundfish, Gulf of Alaska, Pollock, portfolio effects, predation index, predator–prey interactions, synchrony, trophic stability

## Abstract

Predation can have substantial and long‐term effects on the population dynamics of ecologically important prey. Diverse predator assemblages, however, may produce stabilizing (i.e., portfolio) effects on prey mortality when consumption varies asynchronously among predators. We calculated spatiotemporal variation in predation on a dominant forage species to quantify synchrony and portfolio effects in a food web context and better understand diversity–stability relationships in a large marine ecosystem that has undergone considerable changes in community composition. We selected Walleye Pollock (*Gadus chalcogrammus*) as our case study because they support some of the largest, most valuable commercial fisheries in the world and serve as essential prey for an array of economically and culturally important species. Thus, there are sufficient data for Pollock with which to test ecological theories in an empirical setting. Spatially explicit predation indices accounted for annual variation in predator biomass, bioenergetics‐based rations, and age‐specific proportions of Pollock consumed by a suite of groundfishes in the Gulf of Alaska (1990–2015). We found that Arrowtooth Flounder (*Atheresthes stomias*) was, by far, the dominant Pollock predator (proportional consumption: 0.74 ± 0.14). We also found synchronous trends in consumption among predator species, indicating a lack of portfolio effects at the basin scale. This combination of a single dominant predator and synchronous consumption dynamics suggests strong top‐down control over Pollock in the Gulf of Alaska, though the degree of synchrony was highly variable at all spatial scales. Whereas synchrony generally increased in the western subregion, consumption in the central Gulf of Alaska became less synchronous through time. This suggests diminished trophic stability in one area and increased stability in another, thereby emphasizing the importance of spatiotemporal heterogeneity in maintaining food web structure and function. Finally, total Pollock consumption was highly variable (ranging from 1.87 to 7.63 Tg) and often exceeded assessment‐based estimates of productivity. We assert that using our holistic and empirically derived predation index as a modifier of assumed constant natural mortality would provide a practical method for incorporating ecological information into single‐species stock assessments.

## Introduction

Predation has been identified as an important source of mortality for marine fishes, often resulting in far greater losses than those due to fishing (Schoener 1983, Bax 1991, Christensen and Pauly 1993). Although intense predation can have substantial and long‐term effects (Polis et al. 1996, Link 2002, Hixon and Jones 2005), food webs composed of a wide array of consumers may decrease predator control and promote stability in the population dynamics of prey when compared to those that are dominated by few predators. The stabilizing effects of diverse predator assemblages are made possible by asynchronous predator dynamics (e.g., trends in abundance, metabolic rates, prey‐specific consumption), which lessen overall variability in prey mortality (Polis and Strong 1996, Fu et al. 2017, Oken et al. 2018). This type of variance reduction is referred to as the “portfolio effect” (Markowitz 1952, Schindler et al. 2015).

Portfolio effects have been used as a way of understanding diversity–stability relationships in a variety of ecological systems (Hooper et al. 2005). The basic premise is that community dynamics are less variable than the dynamics of component species by way of statistical averaging (Doak et al. 1998). The strength of portfolio effects, therefore, depends on the degree of covariation among individual species (Tilman 1999). Marginal or negative covariation tends to decrease overall variance and, thus, increase stability at the community level (McNaughton 1977). Positive covariation, on the other hand, increases the magnitude of community‐level variation, thereby decreasing both the potential for portfolio effects and ecosystem stability. The strength of portfolio effects also depends upon the spatial and temporal scale at which observations are made, as variation (e.g., in species abundances or environmental conditions) tends to increase from fine to coarse scales (Levin 1992, Hunsicker et al. 2011). From a food web perspective, asynchronous consumption by a diverse assemblage of consumers may decrease variability in the predation pressure experienced by prey (Oken et al. 2018). Depending upon the intensity of predation, this stabilizing condition may take place at relatively high or relatively low prey abundance.

Considerable shifts in community composition have generated questions about predation pressure and trophic stability in the Gulf of Alaska (Anderson and Piatt 1999, Litzow 2006). What was once a demersal fish community dominated by Walleye Pollock (*Gadus chalcogrammus*; i.e., Pollock), a species that supports some of the world’s largest fisheries and serves as important prey for a variety of other stocks (FAO 2014), is now comprised primarily of upper trophic‐level groundfish predators (Anderson and Piatt 1999, Mueter and Norcross 2002). Although this shift in community composition has been attributed, at least in part, to warming temperatures (Anderson and Piatt 1999, Bailey 2000), decreases in prey biomass and concurrent increases in predator abundance (e.g., Litzow and Ciannelli 2007, Dorn et al. 2017) signified a change in complex ecological interactions. Additionally, a number of stock assessment and food web models (e.g*.*, Hollowed et al. 2000b, Aydin et al. 2007, Gaichas et al. 2010, van Kirk et al. 2012, Dorn et al. 2017) have identified predation mortality as an important driver of Pollock biomass within the region. Thus, we were interested in quantifying spatiotemporal variation in predation to better understand the population dynamics of groundfishes in the Gulf of Alaska. We also used the concept of the portfolio effect to quantify diversity–stability relationships in a food web context.

We analyzed standardized survey data to quantify spatial and temporal variation in consumption of Pollock by major groundfish predators in the Gulf of Alaska (1990 to 2015). Time‐varying and spatially explicit indices provide predator‐specific and age‐structured estimates of predation mortality for Walleye Pollock. As such, we assert that these predation indices can provide a relatively simple way of integrating ecological information into single‐species stock assessments (e.g., as a modifier of assumed constant natural mortality; sensu Spencer et al. 2016). In addition to quantifying predation mortality, we inferred diversity of the predator assemblage by comparing species‐specific contributions to overall Pollock consumption. We then calculated synchrony (in terms of predator‐specific consumption) and portfolio effects as a way of assessing food web stability within the groundfish community. Most studies focusing on portfolio effects have addressed temporal correlations among species, yet asynchrony among locations has been identified as a major contributor to ecosystem stability (Thorson et al. 2018). Thus, we calculated synchrony and portfolio effects at four spatial scales: basin, the area encompassed by the stock assessment for Gulf of Alaska Pollock (i.e., west of 140° W longitude), subregions, and statistical areas defined by the International North Pacific Fisheries Commission (INPFC). Spatially explicit metrics also illustrate scale‐dependent impacts on interpretations of predator diversity, community stability, and the potential for top‐down control.

## Methods

### Components of the predation index

**Fig. 1:**
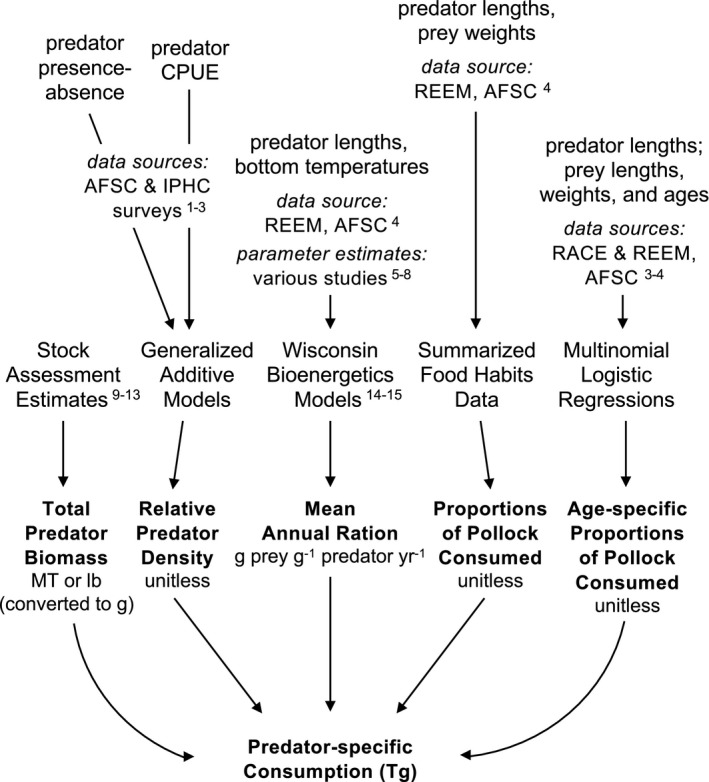
Conceptual diagram of workflow (data sources and analytical methods) used to estimate predator‐, year‐, and area‐specific consumption of Walleye Pollock in the Gulf of Alaska (1990 to 2015). Sources: ^1^Sigler and Lunsford (accessed 2019); ^2^Clark and Hare (2006); ^3^von Szalay and Raring (2016); ^4^Livingston et al. (2017); ^5^Harvey (2009); ^6^Holsman and Aydin (2015); ^7^Holsman et al. 2019; ^8^Holsman et al. (*unpublished data*); ^9^Barbeaux et al. (2017); ^10^Dorn et al. (2017); ^11^Hanselman et al. (2017); ^12^Spies et al. (2017); ^13^Stewart and Hicks (2017); ^14^Kitchell et al. (1977); ^15^Deslauriers et al. (2017).

We used stock assessment‐based estimates of predator biomass, relative predator densities modeled from fishery‐independent survey data, bioenergetics‐based annual rations, and proportional food habits information to calculate consumption of Walleye Pollock by major groundfishes in the Gulf of Alaska (Fig. 1). We calculated time‐varying and spatially explicit indices of predation for young‐of‐the‐year (YOY; 0 yr), juvenile (1 and 2 yr), and adult (3+ yr) Pollock (1990 to 2015; Eq. 1), accounting for five predators: Arrowtooth Flounder (*Atheresthes stomias*; ATF), Pacific Cod (*Gadus chalcogrammus*; PC), Pacific Halibut (*Hippoglossus stenolepis*; PH), Sablefish (*Anoplopoma fimbria*; SBL), and Walleye Pollock (WEP) conspecifics. Cumulatively, predation by these species is thought to make up over 80% of total mortality for Gulf of Alaska Pollock (Gaichas et al. 2015, Dorn et al. 2017). Species‐specific indices of predation were summed across all predators (*S*) to quantify total consumption of Pollock (*P*) age *a* in year *i* and area *j*, as follows:

(1) $$P_{a,i,j}=\sum_{s=1}^{S}P_{s,a,i,j},\;\text{where}\;P_{s,a,i,j}=B_{s,i}\times rD_{s,i,j}\times {\bar{C}}_{s,i,j}\times p_{s,i,j}\times a_{s,i}.$$

*B_s_*_,_ *_i_* represents total biomass of predator *s* in survey year *i*, as estimated in the most recent stock assessment for that species. *rD_s_*_,_ *_i_*_,_ *_j_* is the relative density for predator *s* in year *i* and area *j*, which was used to apportion total predator biomass at finer spatial scales. ${\bar{C}}_{s,i,j}$ denotes the mean annual ration for predator *s* in year *i* and area *j*. When scaled by the first two components of the index (i.e.,* B_s_*_,_ *_i_* and *rD_s_*_,_ *_i_*_,_ *_j_*), ${\bar{C}}_{s,i,j}$ identifies the energetic requirements for each predator species in a given time and place. *p_s_*_,_ *_i_*_,_ *_j_* represents the mean proportion of Pollock observed in the stomachs of predator *s* in year *i* and area *j*. Multiplying the first four terms of the index generates a predator‐specific estimate of Pollock (Tg) consumed in each area and year. Finally, *a_s_*_,_ *_i_* represents the gravimetric proportion of Pollock age class *a* found in the diets of predator *s* in year *i*. This term allows for age‐specific estimates of Pollock consumed. We calculated time‐varying indices of predation in area *j* at the following spatial scales: basin (i.e., entire Gulf of Alaska), the area encompassed by the stock assessment for Gulf of Alaska Pollock (i.e., west of 140° W longitude), subregion (i.e., western, central, eastern Gulf of Alaska), and statistical area (i.e., Shumagin [610], Chirikof [620], Kodiak [630], Yakutat [640], and Southeastern [650]; Appendix S1: Fig. S1). Detailed methods used to estimate each component of the index are described below. All analyses were conducted using the statistical programming environment R (R Core Team 2018). Data sources and script files can be found on Zenodo (see *Data Availability*).

### Total predator biomass, B_s,i_

We compiled estimates of total predator biomass (*B_s_*_,_ *_i_*) from the most recent stock assessment for each species. When combined with other components of the index, total predator biomass scales from individual‐ to population‐level consumption. Stock assessment estimates of *B_s_*_,_ *_i_* pertained to a subset of each stock (i.e., Arrowtooth Flounder ≥ 19 cm, Pacific Cod ≥ 0 cm, Pacific Halibut ≥ 82 cm, Sablefish ≥ 45 cm, and Walleye Pollock ≥ 37 cm), which are referred to as “assessed” fish from here on. Stock assessments for Arrowtooth Flounder (Spies et al. 2017) and Pacific Cod (Barbeaux et al. 2017) encompassed the entire Gulf of Alaska. For Sablefish, we summed subregional estimates of *B_s_*_,_ *_i_* (i.e., western Gulf of Alaska, central Gulf of Alaska, west Yakutat, and east Yakutat/Southeast) to account for the entire basin (Hanselman et al. 2017). The assessment model for Pacific Halibut was developed on a coast‐wide scale, combining the Gulf of Alaska, Eastern Bering Sea, Aleutian Islands, British Columbia (Canada), and U.S. West Coast (Stewart and Hicks 2017). We adjusted Pacific Halibut *B_s_*_,_ *_i_* by multiplying coast‐wide estimates by the proportion of fish ≥32 in (82 cm) caught in International Pacific Halibut Commission (IPHC) regulatory areas 4A, 3B, 3A, and 2C (IPHC setline survey, 1998 to 2015). Additionally, the coast‐wide assessment did not estimate Pacific Halibut *B_s_*_,_ *_i_* prior to 1996. Thus, we back‐calculated *B_s_*_,_ *_i_* for 1990 and 1993 based on biomass trends from the Alaska Fisheries Science Center (AFSC) bottom trawl survey (methods described by Barnes et al. 2018), which were highly correlated with trends from the IPHC setline survey (Pearson; *r* = 0.905, *t*_7_ = 5.622, *P* < 0.001). Walleye Pollock were assessed separately for the areas west and east of 140° W, referred to as the Gulf of Alaska and Southeast Alaska, respectively (Dorn et al. 2017). We summed biomass estimates from these two areas to approximate *B_s_*_,_ *_i_* for Walleye Pollock at the basin scale. All *B_s_*_,_ *_i_* estimates were converted to grams before being incorporated into predation indices.

### Relative predator densities, rD_s,i,j_

We used standardized survey data to estimate relative predator densities throughout the Gulf of Alaska. Bottom trawl survey data collected by the AFSC’s Resource Assessment and Conservation Engineering (RACE) Division were used to estimate relative predator densities (*rD_s_*_,_ *_i_*_,_ *_j_*) for Arrowtooth Flounder, Pacific Cod, and Walleye Pollock (Appendix S1: Fig. S1). We used setline and longline survey data to estimate *rD_s_*_,_ *_i_*_,_ *_j_* for Pacific Halibut and Sablefish (Appendix S1: Fig. S1) because these gear types more effectively sample larger (i.e., older) individuals of these species. This was an important consideration, given that *B_s_*_,_ *_i_* obtained from stock assessment models correspond to 8+ yr (≥81 cm) Pacific Halibut (Stewart and Hicks 2017) and 2+ yr (≥45 cm) Sablefish (Hanselman et al. 2017). These size and age ranges are also most likely to consume fish such as Walleye Pollock (Yang 1995, Harvey 2009). Additional information about survey designs and data collection can be found in Supporting Information (Appendix S1).

To match the component of the population encompassed by *B_s_*_,_ *_i_* and that used to estimate *rD_s_*_,_ *_i_*_,_ *_j_*, we adjusted haul‐ or station‐specific catch per unit effort (CPUE; kg × ha^‐1^) to include only assessed fish (i.e., Arrowtooth Flounder ≥ 19 cm, Pacific Cod ≥ 0 cm, Pacific Halibut ≥ 82 cm, Sablefish ≥ 45 cm, and Walleye Pollock ≥ 37 cm). To adjust CPUE, we first estimated the mass of each measured fish using known length–mass relationships identified in the Arrowtooth Flounder, Pacific Cod, and Sablefish stock assessments (Appendix S1: Table S1). We used the bias‐corrected method described by Brodziak (2012) to quantify the length–mass relationship for Walleye Pollock. There was no need to calculate mass or adjust CPUE for Pacific Halibut because station‐specific estimates of ≥32 in (82 cm) were provided by IPHC. For each species, we calculated total mass (e.g., all Arrowtooth Flounder sampled) and the mass of assessed individuals (e.g., Arrowtooth Flounder ≥ 19 cm) for each haul or station. We then used the ratio of these two metrics as a multiplier of haul‐ or station‐specific CPUE.

We used delta (i.e., hurdle) models to quantify species‐specific probability of occurrence and log‐transformed CPUE for positive catches (sensu Barnes et al. 2018). Presence–absence and log‐transformed CPUE were modeled as a function of survey year and splines of location (latitude and longitude) and depth. Bottom temperatures were available from bottom trawl surveys and were included as an additional covariate for Arrowtooth Flounder, Pacific Cod, and Walleye Pollock models. Temperature data were only available during the latter portions of the IPHC setline and AFSC longline surveys. Thus, temperature was excluded from models pertaining to Pacific Halibut and Sablefish. For computational efficiency, we first ran generalized additive models (GAMs) with all possible combinations of the component terms (mgcv package, Wood 2011; MuMIn package, Bartoń 2017) and used ΔAIC to identify best‐fit GAMs. We then re‐ran best‐fit GAMs as generalized additive mixed models (GAMMs) with and without a Gaussian spatial autocorrelation structure for each survey year. Only GAMMs with spatial autocorrelation that resulted in an improved fit (based on the change in the Akaike information criterion, ΔAIC) were selected over best‐fit GAMs.

We used best‐fit models to quantify year‐specific probabilities of occurrence (PO) and predicted abundances (PA) across a 50 × 50 km uniform grid spanning the study area. We then multiplied ${\text{PO}}_{s_{i,g}}$ and ${\text{PA}}_{s_{i,g}}$ for species *s* in year *i* and grid cell *g* to estimate predator density (*D_s_*_,_ *_i_*_,_ *_g_*). We calculated relative predator densities (*rD_s_*_,_ *_i_*_,_ *_g_*) by dividing *D_s_*_,_ *_i_*_,_ *_g_* by the sum of all grid cells sampled in year *i*: $rD_{s,i,g}=D_{s,i,g}/\sum_{g=1}^{G}D_{s,i,g}$, where *G* is the total number of grid cells *g* sampled in year *i*. The total number of grid cells varied among species, but was constant across years ( $G_{{\text{ATF}}_{i}}$ = 195, $G_{{\text{PC}}_{i}}$ = 195, $G_{{\text{PH}}_{i}}$ = 157, $G_{{\text{SBL}}_{i}}$ = 72, and $G_{{\text{WEP}}_{i}}$ = 195). We summed *rD_s_*_,_ *_i_*_,_ *_g_* within each statistical area to quantify *rD_s_*_,_ *_i_*_,_ *_j_* at intermediate spatial scales. For the subregion scale, we recategorized the Shumagin statistical area (610) as the western Gulf of Alaska, summed *rD_s_*_,_ *_i_*_,_ *_g_* estimates in the Chirikof (620) and Kodiak (630) statistical areas to represent the central Gulf of Alaska, and summed *rD_s_*_,_ *_i_*_,_ *_g_* in the Yakutat (640) and Southeastern (650) statistical areas to represent the eastern Gulf of Alaska. These subregions are consistent with definitions used by the AFSC (e.g., Aydin et al. 2007, Dorn et al. 2017). Finally, we summed *rD_s_*_,_ *_i_*_,_ *_g_* within the area encompassed by the stock assessment for Gulf of Alaska Pollock (i.e., the area west of 140**° W** longitude). There were no IPHC setline survey data prior to 1998. Thus, we assigned area‐specific mean densities for Pacific Halibut in 1990, 1993, and 1996 using the available time series (1998–2017). When multiplied by *B_s_*_,_ *_i_*, *rD_s_*_,_ *_i_*_,_ *_j_* provides an estimate of predator biomass in each year and area of interest.

### Mean annual rations, ${\bar{C}}_{s,i,j}$

We used Wisconsin bioenergetics models (Kitchell et al. 1977, Deslauriers et al. 2017) to calculate maximum daily consumption rates (*C*_max_; g·g^−1^·d^−1^) for assessed fish. *C*_max_ was estimated as a function of individual predator mass (*W*) and haul‐specific temperature (*T_h_*; Hanson et al. 1997, Holsman and Aydin 2015) such that

(2) $$C_{\max}=C_{A}\times W^{\left(C_{B}\right)}\times f\left(T_{h}\right),\;\text{where}$$

$$f\left(T_{h}\right)=V^{X}\times e^{X\left(1-V\right)},$$

$$V=\frac{T_{C_{M}}-T_{h}}{T_{C_{M}}-T_{C_{0}}},\;X=\frac{Z^{2}\times {\left(1+{\left(1+\frac{40}{Y}\right)}^{0.5}\right)}^{2}}{400},\;\text{and}$$

$$Z=\log\left(C_{Q}\right)\times \left(T_{C_{M}}-T_{C_{0}}\right),\;Y=\log\left(C_{Q}\right)\times \left(T_{C_{M}}-T_{C_{0}}+2\right).$$

*C_A_* and *C_B_* are the intercept and slope for the allometric consumption equation based on predator mass (kg) and are scaled by temperature *f*(*T_h_*). $T_{C_{M}}$ represents the temperature threshold above which consumption ceases, $T_{C_{0}}$ is the temperature where consumption rates are highest, and *C_Q_* approximates the rate of increase in consumption at low temperatures. Bioenergetics model parameters were sourced from Holsman and Aydin (2015), Holsman et al. (2019), Holsman et al., *unpublished data*, and Harvey (2009) (Table 1).

**Table 1:** Parameters from bioenergetics models that were used to estimate maximum daily consumption (g·g^−1^·d^−1^) for each Pollock predator (ATF, Arrowtooth Flounder; PC, Pacific Cod; PH, Pacific Halibut; SBL, Sablefish; WEP, Walleye Pollock). C_A_ and C_B_ are the intercept and slope for the allometric consumption equation. C_Q_ approximates the rate of increase in consumption at low temperatures, T_C0_ is the temperature where consumption rates are highest, and T_CM_ represents the temperature threshold above which consumption ceases.

Parameter	ATF^†^	PC^‡^	PH^§^	SBL^¶^	WEP^†^
*C_A_*	0.1250	0.0350	0.0625	0.4200	0.1190
*C_B_*	–0.1990	–0.1220	–0.1076	–0.3300	–0.4600
*C_Q_*	2.497	3.079	3.084	2.200	2.600
$T_{C_{0}}$	20.512	10.957	12.970	18.000	10.000
$T_{C_{M}}$	26.000	25.901	18.000	23.000	15.000
*D*_juveniile_	346 (<40 cm)	365 (<55 cm)	365^#^ (<82 cm)	365^#^ (<45 cm)	365 (<40 cm)
*D*_adult_	306 (≥40 cm)	329 (≥5 cm)	365^#^ (≥82 cm)	365^#^ (≥45 cm)	365 (≥40 cm)

Notes

Mean estimated foraging days (*D*_juveniile_ or *D*_adult_) are also listed, with size ranges used to categorize fish as juveniles or adults (parentheses). Superscripts indicate sources of information.

† Holsman and Aydin (2015)

‡ Holsman et al. (*unpublished data*)

§ Holsman et al. (2019)

¶ Harvey (2009)

# Assumed values

Individual masses were only measured for predators subsampled for food habits. Additionally, temperature data were unavailable for much of the IPHC setline and AFSC longline time series. Thus, we calculated maximum daily consumption using data from the AFSC bottom trawl survey. Temperature data were missing in the Shumagin statistical area in 1990, so *C*_max_ estimates were assumed to be the same as those in Shumagin in 1993. Additionally, we assumed area‐specific mean *C*_max_ for Sablefish after 2011 because this species was not subsampled for gut contents in 2013 or 2015. We multiplied *C*_max_ by the estimated number of foraging days per year (Holsman and Aydin 2015; Holsman et al., *unpublished data*; Holsman et al. 2019) to scale from maximum daily consumption (g·g^−1^·d^−1^) to maximum annual consumption (g·g^−1^·yr^−1^, Table 1). We then calculated mean annual rations for species *s* in year *i* and area *j* ( ${\bar{C}}_{s,i,j}$) to include in predation indices. Year‐ and area‐specific energetic requirements of each predator “population” were estimated by multiplying *B_s_*_,_ *_i_*, *rD_s_*_,_ *_i_*_,_ *_j_*, and ${\bar{C}}_{s,i,j}$.

### Proportions of Pollock consumed, p_s,i,j_

We used food habits data collected by the AFSC’s Resource Ecology and Ecosystem Modeling (REEM) Program to quantify proportions of Pollock in the diets of assessed predators. Food habits data were unavailable from the IPHC setline and AFSC longline surveys due to high rates of regurgitation that result from prolonged fishing (I. Stewart, *personal communication*). Therefore, dietary analyses were based solely on data from the AFSC bottom trawl survey. Subsampling methods and additional information about food habits data collection can be found in Supporting Information (Appendix S1).

As a result of size‐structured subsampling, fork lengths of fish selected for stomach content analyses were not representative of the overall catch. To correct for this, we weighted food habits data according to the length composition of predators caught. First, we defined 10‐cm fork length bins *b* for each predator species *s*. We then calculated the proportion of fish caught or sampled in length bin *b* during haul *h*. Proportions were calculated for all fish caught during the bottom trawl survey $\left(P_{T_{s,b,h}}=N_{T_{s,b,h}}/\sum_{b=1}^{B}N_{T_{s,h}}\right)$ and only those fish subsampled for food habits $\left(P_{F_{s,b,h}}=N_{F_{s,b,h}}/\sum_{b=1}^{B}N_{F_{s,h}}\right)$. *N* denotes the total number of fish caught or subsampled for species *s* and *B* represents the total number of length bins observed in a particular haul. We calculated length‐based weighting factors (WF*_L_*) as follows: ${\text{WF}}_{L_{s,b,i,h}}=P_{T_{s,b,h}}/P_{F_{s,b,h}}$. By multiplying raw food habits data and length‐based weighting factors, predators that were over‐represented in the food habits database (e.g., if the haul‐specific proportion of Sablefish measuring 45–54 cm was 0.35 for subsamples, but only 0.27 from the overall catch) would be down‐weighted and predators that were under‐represented in the food habits database (e.g., the haul‐specific proportion of Sablefish measuring 45–54 cm was 0.35 for subsamples, but 0.52 from the overall catch) would be weighted more heavily.

In addition to size‐structured subsampling, survey effort was not proportional to predator biomass. Thus, biomass weighting was necessary to scale up from individual diets. We calculated biomass weighting factors (WF*_B_*) by dividing the predicted density for species *s* in year *i* and grid cell *g* (*D_s_*_,_ *_i_*_,_ *_g_*; described in *Relative predator densities,* *rD_s_*_,_ *_i_*_,_ *_g_*) by the mean predicted density of species *s* in year *i* ( ${\bar{D}}_{s,i}$): ${\text{WF}}_{B_{s,i,g}}=D_{s,i,g}/{\bar{D}}_{s,i}$. As with length‐based weighting factors, food habits data from predators that were over‐represented in the food habits database (e.g., if the proportion of Sablefish subsampled from grid cell *g* was 0.12, but only 0.08 of Sablefish biomass was found in grid cell *g* that year) would be down‐weighted and vice versa. We then multiplied the mass of each prey taxon *q* observed in predator stomach *r* (*w_q_*_,_ *_r_*) by fork length and biomass weighting factors: $w_{w_{q,r}}=w_{q,r}\;\times \;{\text{WF}}_{L}\;\times \;{\text{WF}}_{B}$ (species *s*, year *i*, and location [haul *h* or grid cell *g*]; subscripts were removed for simplicity). We used fork length‐ and biomass‐weighted prey masses ( $w_{w_{q,r}}$) to estimate the proportion of prey consumed (*p_q_*) by predator species *s* in year *i* and area *j*:

(3) $$p_{q}=\frac{\sum_{r=1}^{R}w_{w_{q,r}}}{\sum_{r=1}^{R}\sum_{q=1}^{Q}w_{w_{q,r}}},$$

where *Q* represents the total number of prey taxa and *R* is the total number of predator stomachs observed (Chipps and Garvey 2007). Proportions of Pollock, termed *p_s_*_,_ *_i_*_,_ *_j_*, were included in predation indices. We assumed mean *p_s_*_,_ *_i_*_,_ *_j_* for Sablefish in 2013 and 2015, when no diet data were collected.

### Age compositions of Pollock as prey, a_s,i_

The stock assessment for Gulf of Alaska Pollock accounts for age‐specific natural mortality (Dorn et al. 2017). Thus, we were interested in quantifying age‐specific predation mortality. First, we used all available bottom trawl survey data to quantify age–length (von Bertalanffy 1938) and bias‐corrected length–mass (Brodziak 2012) relationships for Pollock in the Gulf of Alaska. We then used parameters from these relationships to estimate ages and masses of Pollock found in predator stomachs. Due to variable stages of digestion, only a subset of Pollock was measured (standard length, mm). From these, we used multinomial logistic GAMs (VGAM package in R; Yee 2015) to estimate mean proportions of age‐0, age‐1, age‐2, and age‐3+ Pollock consumed by species *s* in year *i* (*a_s_*_,_ *_i_*). Small sample sizes precluded spatially explicit estimates of Pollock age. Measurable Pollock were not observed in the diets of Sablefish in 2005, 2013, or 2015, or in the diets of Walleye Pollock in 2005, 2011, or 2015. For these species and years, we assigned mean proportions across all other years.

### Predation indices, P_a,i,j_, synchrony, and portfolio effects

We calculated age‐specific consumption of Walleye Pollock (*P_a_*_,_ *_s_*_,_ *_i_*_,_ *_j_*; Eq. 1) at the following spatial scales: basin, the area encompassed by the stock assessment for Gulf of Alaska Pollock, subregion, and statistical area. We summed predator‐specific indices to estimate “total” consumption on Pollock in each survey year. Data limitations precluded estimates in the eastern Gulf of Alaska between 1996 and 2001, in the Yakutat statistical area from 1996 to 2001, and in the Southeastern statistical area prior to 2005. We quantified predator contributions to Pollock predation mortality by dividing *P_a_*_,_ *_s,i_*_,_ *_j_* by *P_a_*_,_ *_i_*_,_ *_j_*.

We also calculated variance ratios, a measure of correlation among multivariate responses, to assess the degree of synchrony in consumption among Pollock predators (Loreau and de Mazancourt 2008, Gonzalez and Loreau 2009, Oken et al. 2018). Variance ratios (VR*_i_*_,_ *_j_*) were computed using a five‐year moving window such that the degree of synchrony in 1990 represented consumption dynamics during the proceeding five survey years (i.e., 1990, 1993, 1996, 1999, and 2001). The duration and frequency of standardized bottom trawl surveys prevented longer (e.g., 10‐yr) moving windows and variance ratios after 2007. ${\text{VR}}_{i,j}=\text{var}\left(\sum P_{s,i,j}\right)/\sum \text{var}\left(P_{s,i,j}\right)$, where $\text{var}(\sum P_{s,i,j})$ represents the variance of total Pollock consumption (all predators combined) in window *i* and area *j* and $\sum \text{var}(P_{s,i,j})$ is the sum of predator‐specific variances in consumption in window *i* and area *j*. VR*_i_*_,_ *_j_* is equal to one when consumption is, on average, statistically independent among predators (i.e., overall variance is equivalent to the sum of predator‐specific variances). VR*_i_*_,_ *_j_* > 1  (i.e., the sum of predator‐specific variances is less than the variance of total consumption) indicates synchronous trends in consumption among species and VR*_i_*_,_ *_j_* < 1 (i.e., the sum of predator‐specific variances is greater than the variance of total consumption) indicates asynchronous predator dynamics.

The degree of portfolio effects (PE*_j_*) was estimated as 1 − VR*_i_*_,_ *_j_* (sensu Thorson et al. 2018). We used PE*_i_*_,_ *_j_* to make inferences about trophic stability, specifically related to the variation in predation pressure experienced by Walleye Pollock. Greater PE*_i_*_,_ *_j_* reflected greater trophic stability and lower PE*_i_*_,_ *_j_* suggested lower trophic stability. We computed Pearson’s correlation matrices to estimate species‐specific correlations in Pollock consumption. We explored spatiotemporal anomalies in consumption by dividing species‐, subregion‐, and year‐specific consumption by species‐specific means for the entire Gulf of Alaska and time series. To understand how predation mortality compared to estimates of Pollock productivity, we calculated year‐specific ratios of age‐3+ consumption to total Pollock biomass within the area encompassed by the stock assessment (Dorn et al. 2017).

## Results

### Components of the predation index

Arrowtooth Flounder biomass (*B_s_*_,_ *_i_*) increased from 1990 to 2005 and decreased thereafter (Table 2). Walleye Pollock showed an opposing trend, though with greater interannual variability. Pacific Cod and Pacific Halibut *B_s_*_,_ *_i_* declined throughout the time series. There was no clear temporal trend in Sablefish *B_s_*_,_ *_i_*.

**Table 2:** Total biomass estimates (*B_s_*_,_ *_i_*, Mg) from the most recent stock assessments for Arrowtooth Flounder (ATF), Pacific Cod (PC), Pacific Halibut (PH), Sablefish (SBL), and Walleye Pollock (WEP) in the Gulf of Alaska (1990 to 2015).

Year	ATF† 1+ yr; ≥ 19 cm	PC‡ 0+ yr; ≥ 0 cm	PH§ 8+ yr; ≥ 82 cm	SBL¶ 2+ yr; ≥ 45 cm	WEP# 3+ yr; ≥ 37 cm	
					GOA	SE
1990	1,660,800	583,841	1993 × 0.706	251,000	1,479,000	26,101
1993	1,773,450	516,782	1996 × 1.045	261,000	1,748,000	12,337
1996	1,770,270	429,292	799,683 (0.782)	200,000	1,013,000	75,596
1999	1,835,310	320,235	726,201 (0.847)	183,000	737,000	31,836
2001	1,957,130	286,165	583,773 (0.798)	182,000	625,000	28,979
2003	2,035,310	292,752	528,888 (0.810)	202,000	1,021,000	26,658
2005	2,069,910	247,481	432,273 (0.853)	197,000	713,000	36,901
2007	2,054,040	246,629	406,418 (0.838)	183,000	580,000	41,075
2009	1,962,540	307,285	351,987 (0.730)	164,000	1,170,000	47,885
2011	1,826,620	345,269	319,782 (0.742)	181,000	1,330,000	66,969
2013	1,701,770	316,926	339,740 (0.742)	157,000	1,277,000	39,879
2015	1,571,460	312,414	301,639 (0.740)	140,000	1,771,000	26,173

Notes

Ages (yr) and lengths (cm) encompassed within total biomass estimates are also shown. Species‐specific references are indicated as superscripts. The stock assessment for Pacific Halibut was conducted on a coast‐wide basis and included total biomass estimates from 1996 onward. Thus, total halibut biomass was back‐calculated for 1990 and 1993 using trends in predicted biomass from the Alaska Fisheries Science Center bottom trawl survey. Numbers in parentheses denote biomass scalars for Pacific Halibut (i.e., proportions ≥ 32 in or 82 cm caught in International Pacific Halibut Commission regulatory areas 4A, 3B, 3A, and 2C during the IPHC setline survey). The stock assessment for Walleye Pollock was partitioned at 140° W longitude, with the Gulf of Alaska (GOA) portion to the west and the Southeastern (SE) portion to the east.

† Spies et al. (2017)

‡ Barbeaux et al. (2017)

§ Stewart and Hicks (2017)

¶ Hanselman et al. (2017)

# Dorn et al. (2017)

We found that full models best described the distributions and abundances of each predator species (Appendix S1: Table S2; Appendix S1: Figs. S2, S3). Accounting for spatial autocorrelation improved the fit of all models except presence–absence for Walleye Pollock and CPUE for Sablefish (Appendix S1: Tables S3 and S4). GAMMs did not converge when modeling presence–absence of Pacific Halibut. Because Sablefish were observed at nearly all stations encompassed within the AFSC longline survey, we did not separately model presence–absence for this species. Relative predator densities (*rD_s_*_,_ *_i_*_,_ *_j_*) for Arrowtooth Flounder, Pacific Cod, and Walleye Pollock were highest in the western and central subregions, whereas *rD_s_*_,_ *_i_*_,_ *_j_* for Pacific Halibut and Sablefish were more evenly distributed throughout the study area (Appendix S1: Fig. S4).

**Fig. 2:**
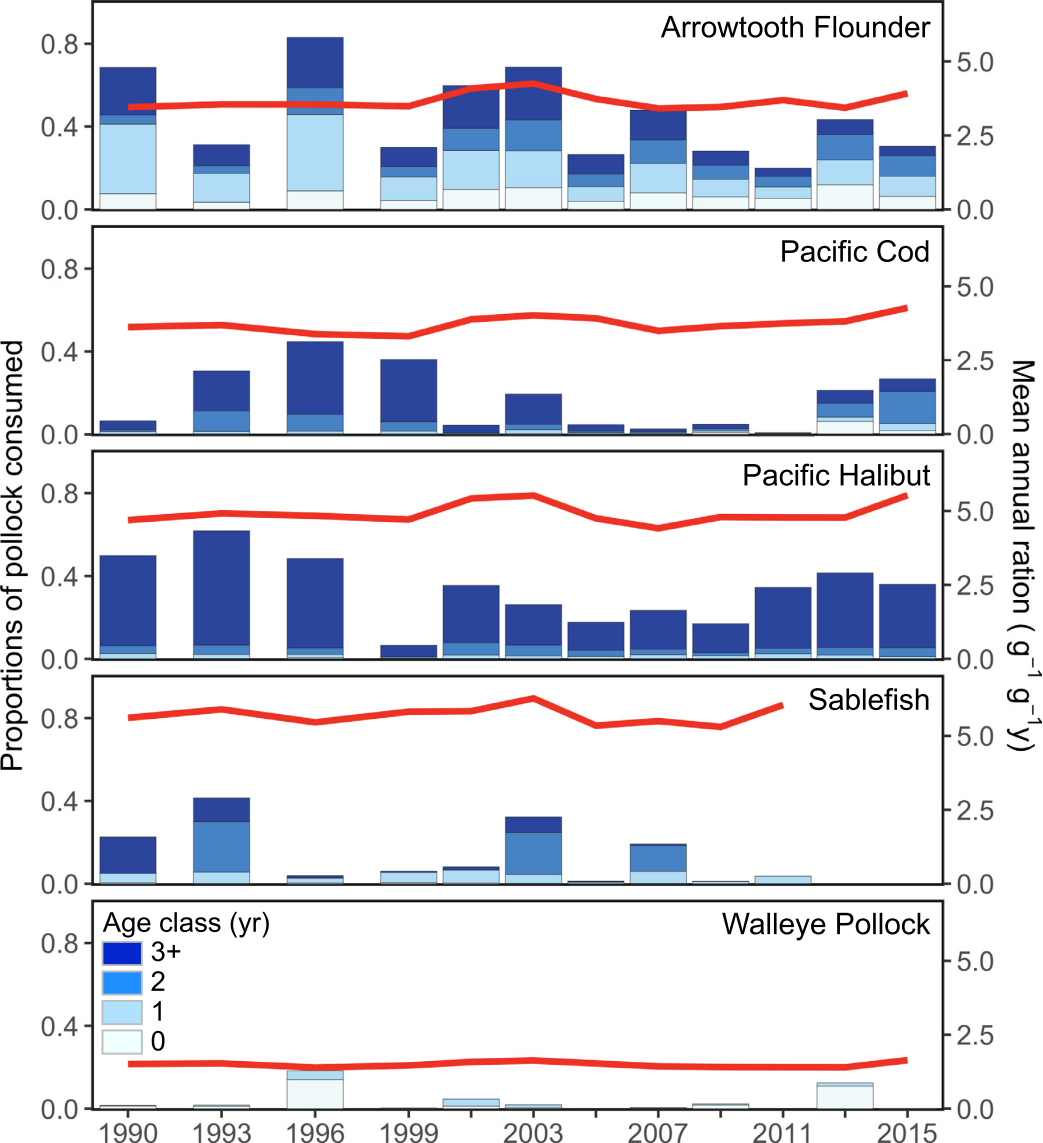
Year‐ and age‐specific proportions of Pollock consumed (*p_s_*_,_ *_i_*_,_ *_j_*; blue bars) and mean annual rations ( ${\bar{C}}_{s,i,j}$, g^−1^·g^−1^·yr^−1^; red lines) by predator and survey year (Gulf of Alaska, 1990–2015). Errors bars for mean annual rations have been excluded for clarity.

Sablefish and Pacific Halibut exhibited the highest mean annual rations ( ${\bar{C}}_{s,i,j}$) of all predators examined (5.7 ± 0.32 and 4.9 ± 0.36 [mean ± SD], respectively) (Fig. 2). ${\bar{C}}_{s,i,j}$ for Arrowtooth Flounder (3.7 ± 0.28) and Pacific Cod (3.7 ± 0.27) were similar to one another, and Walleye Pollock had the lowest ${\bar{C}}_{s,i,j}$ of any species (1.5 ± 0.09). Despite relative differences, temporal trends in ${\bar{C}}_{s,i,j}$ were similar among predators, with a peak in 2003 and relatively little variation throughout the remainder of the time series.

Food habits data showed that Arrowtooth Flounder consumed the greatest proportions of Pollock (*p_s_*_,_ *_i_*_,_ *_j_*, 0.45 ± 0.20), which were a mixture of adult and juvenile fish (age‐3+, 0.38 ± 0.09; age‐2, 0.26 ± 0.11; age‐1, 0.30 ± 0.12; Fig. 2 and Fig. S5). Pollock also made up a large proportion of Pacific Halibut diets (0.33 ± 0.16). The relatively large Pacific Halibut that we analyzed fed primarily on adults (age‐3+, 0.84 ± 0.04). Pacific Cod and Sablefish consumed proportionally less Pollock overall (0.17 ± 0.16 and 0.14 ± 0.13, respectively). Adults were most common in the diets of Pacific Cod (0.58 ± 0.21), whereas juveniles were more common in Sablefish diets (0.74 ± 0.20). We found that Walleye Pollock consumed relatively few conspecifics (0.04 ± 0.06), which were either age‐0 (0.62 ± 0.23) or age‐1 (0.38 ± 0.23) fish.

### Predation indices, P_a,i,j_, synchrony, and portfolio effects

**Fig. 3:**
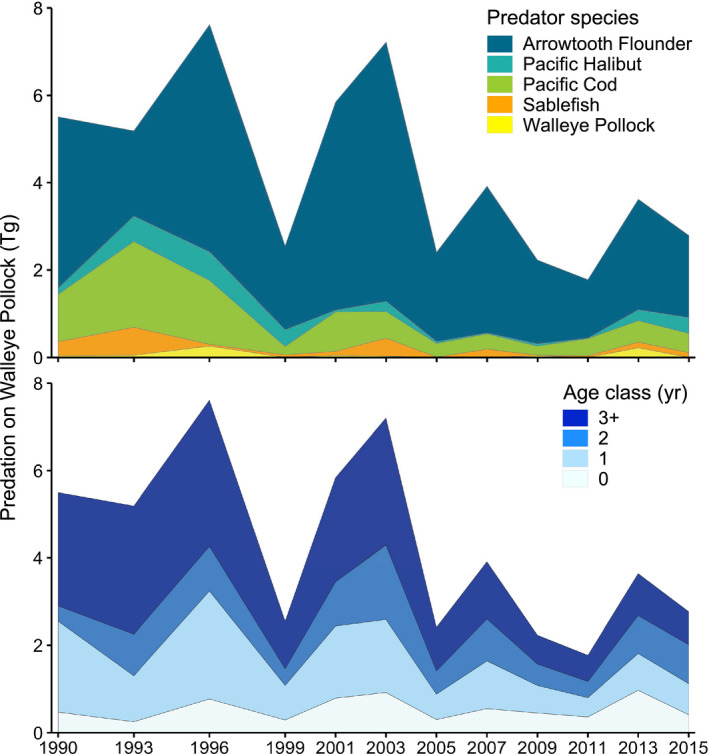
Total consumption of Walleye Pollock (Tg) in the Gulf of Alaska, by predator (top; *P_s_*_,_ *_i_*_,_ *_j_*), survey year (1990–2015), and Pollock age class (bottom; *P_a_*_,_ *_i_*_,_ *_j_*). Predator‐specific indices group all age classes of Pollock. Age‐specific indices include consumption by all groundfish predators combined.

**Fig. 4:**
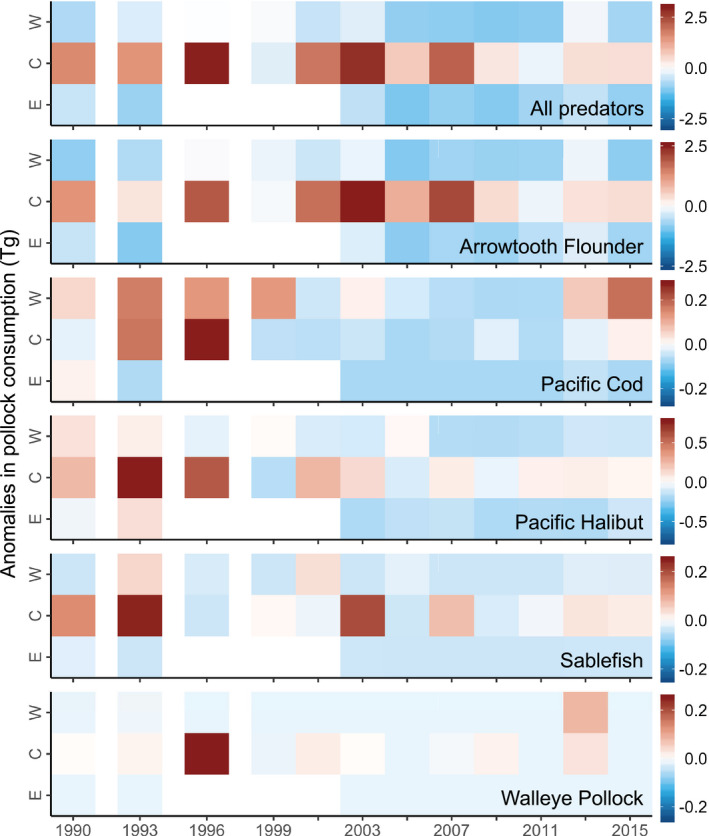
Year‐specific anomalies in Pollock consumption (relative to the Gulf of Alaska mean), by predator and subregion (W, western; C, central; E, eastern Gulf of Alaska). Positive anomalies (Tg) are shown in red and negative anomalies (Tg) are shown in blue. There were no estimates for Pollock predation in the eastern Gulf of Alaska between 1996 and 2001.

**Fig. 5:**
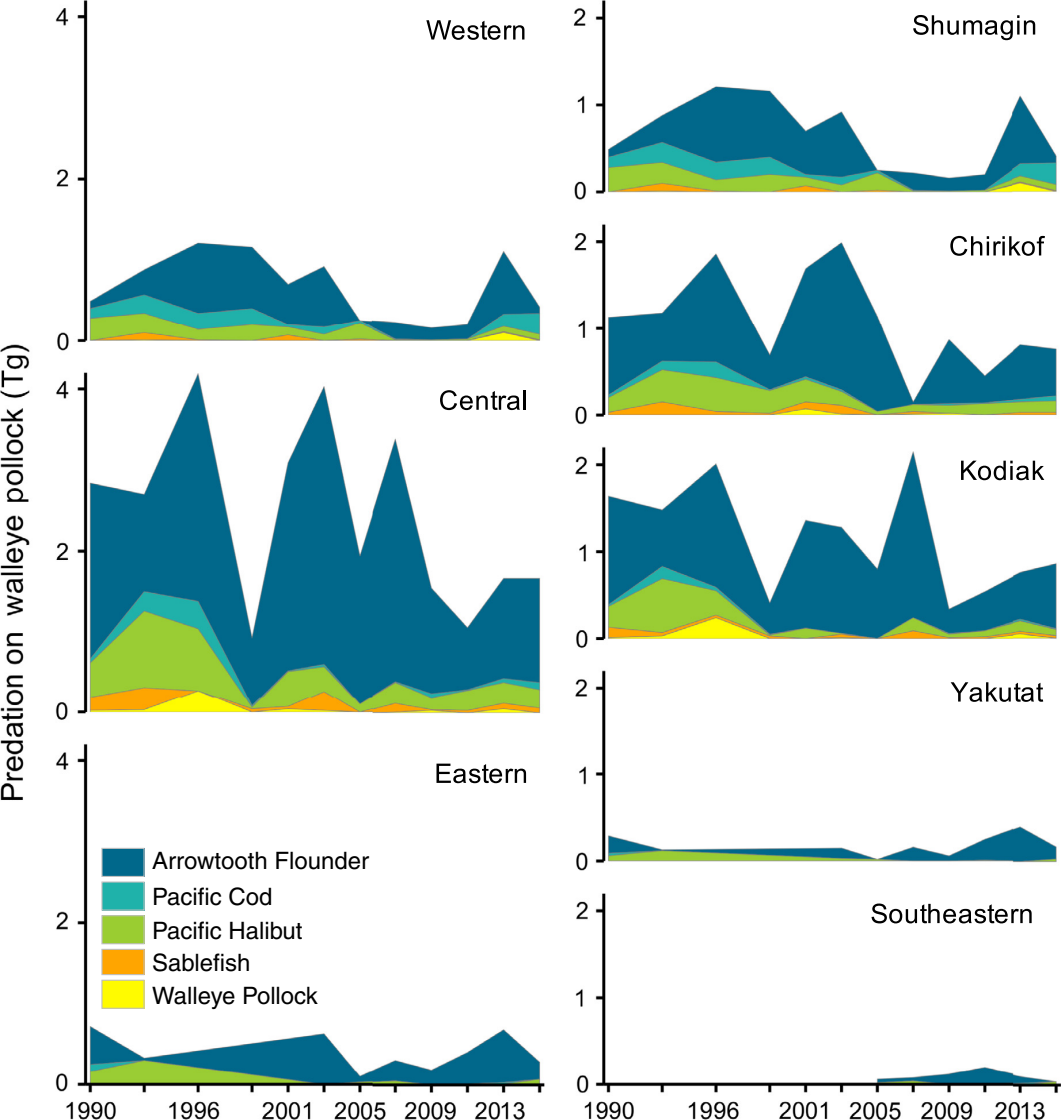
Total consumption of Walleye Pollock (*P_s_*_,_ *_i_*_,_ *_j_*, Tg) in the Gulf of Alaska, by survey year (1990–2015), predator, subregion (left), and International North Pacific Fisheries Commission (INPFC) statistical area (right). There were no estimates for Pollock predation in the eastern Gulf of Alaska between 1996 and 2001, in the Yakutat INPFC statistical from 1996 to 2001, or in the Southeastern statistical area prior to 2005.

Total Pollock consumption (*P_a_*_,_ *_i_*_,_ *_j_*) ranged from 1.87 to 7.63 Tg in the Gulf of Alaska and generally declined throughout the time series. Arrowtooth Flounder were responsible for the vast majority of predation, followed by Pacific Halibut, Pacific Cod, Sablefish, and Walleye Pollock (Table 3; Fig. 3A). Most Pollock prey were age‐3+ adults (0.386 ± 0.089), followed by age‐1 (0.272 ± 0.048) and age‐2 (0.199 ± 0.065) juveniles (Fig. 3B). Relatively few (0.141 ± 0.059) young‐of‐the‐year Pollock were observed. *P_a_*_,_ *_i_*_,_ *_j_* was greatest in 1996 and 2003, with subsequent peaks in 2007 and 2013 (Fig. 3). Each peak was followed by a considerable decrease in *P_a_*_,_ *_i_*_,_ *_j_* that coincided with decreases in proportions of Pollock consumed by Arrowtooth Flounder (Figs. 2, 3). Additionally, spatiotemporal anomalies in Pollock predation closely resembled anomalies in consumption by Arrowtooth Flounder (Fig. 4). We found the greatest amount of Pollock consumption in the central Gulf of Alaska, which was approximately evenly distributed between the Chirikof and Kodiak statistical areas (Fig. 5).

**Table 3:** Species‐specific contributions to Pollock predation mortality in the Gulf of Alaska.

Spatial Scale and/or Location	ATF	PC	PH	SBL	WEP
Basin	0.74 ± 0.14	0.06 ± 0.05	0.16 ± 0.09	0.04 ± 0.03	0.01 ± 0.02
Pollock assessment area	0.76 ± 0.14	0.06 ± 0.06	0.14 ± 0.08	0.03 ± 0.03	0.01 ± 0.02
Western subregion	0.59 ± 0.32	0.16 ± 0.16	0.21 ± 0.23	0.03 ± 0.04	0.01 ± 0.03
Central subregion	0.79 ± 0.14	0.03 ± 0.03	0.14 ± 0.09	0.03 ± 0.03	0.01 ± 0.02
Eastern subregion	0.77 ± 0.29	0.02 ± 0.04	0.21 ± 0.28	0.00 ± 0.01	0.00 ± 0.00
Shumagin statistical area	0.59 ± 0.32	0.16 ± 0.16	0.22 ± 0.23	0.03 ± 0.04	0.01 ± 0.03
Chirikof statistical area	0.69 ± 0.20	0.04 ± 0.03	0.22 ± 0.14	0.05 ± 0.06	0.01 ± 0.02
Kodiak statistical area	0.81 ± 0.15	0.02 ± 0.03	0.12 ± 0.11	0.03 ± 0.02	0.02 ± 0.04
Yakutat statistical area	0.71 ± 0.39	0.01 ± 0.03	0.28 ± 0.39	0.00 ± 0.01	0.00 ± 0.00
Southeastern statistical area	0.66 ± 0.38	0.03 ± 0.07	0.31 ± 0.41	0.00 ± 0.00	0.00 ± 0.00

Notes

Values indicate mean proportions ± SD of total consumption for each predator species within a given area (1990 to 2015). The Yakutat and Southeastern statistical areas include survey years between 2005 and 2015 only.

**Fig. 6:**
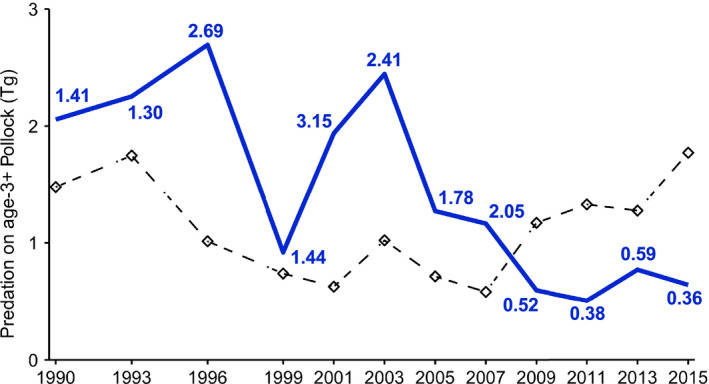
Total consumption (*P_i_*_,_ *_j_*, Tg) of age‐3+ Pollock (solid blue line) within the area encompassed by the Gulf of Alaska stock assessment (i.e., Shumagin, Chirikof, Kodiak, and Yakutat statistical areas), 1990 to 2015. Estimates of total Pollock biomass from the most recent stock assessment are also shown (*B_s_*_,_ *_i_*, Tg; diamonds and dashed line; Dorn et al. 2017). Numbers indicate the ratio of consumption to biomass in a given survey year.

There was no correlation between assessment‐based estimates of Pollock biomass and age‐3+ consumption when analyzing the entire time series (1990 to 2015; *r*_10_ = −0.074, *P* = 0.820) or when isolating the early time period (1990 to 2003; *r*_4_ = 0.371, *P* = 0.469). A negative correlation, however, was evident during the later time period (2005 to 2015; *r*_4_ = −0.837, *P* = 0.038). Consumption of age‐3+ fish exceeded estimates of total biomass between 1996 and 2007 (Fig. 6; Dorn et al. 2017). Consumption‐to‐biomass ratios (*P_a_*_,_ *_i_*_,_ *_j_*:*B_s_*_,_ *_i_*) ranged from 0.36 in 2015 to 3.15 in 2001. *P_a_*_,_ *_i_*_,_ *_j_* was less than Pollock *B_s_*_,_ *_i_* from 2009 to 2015.

**Fig. 7:**
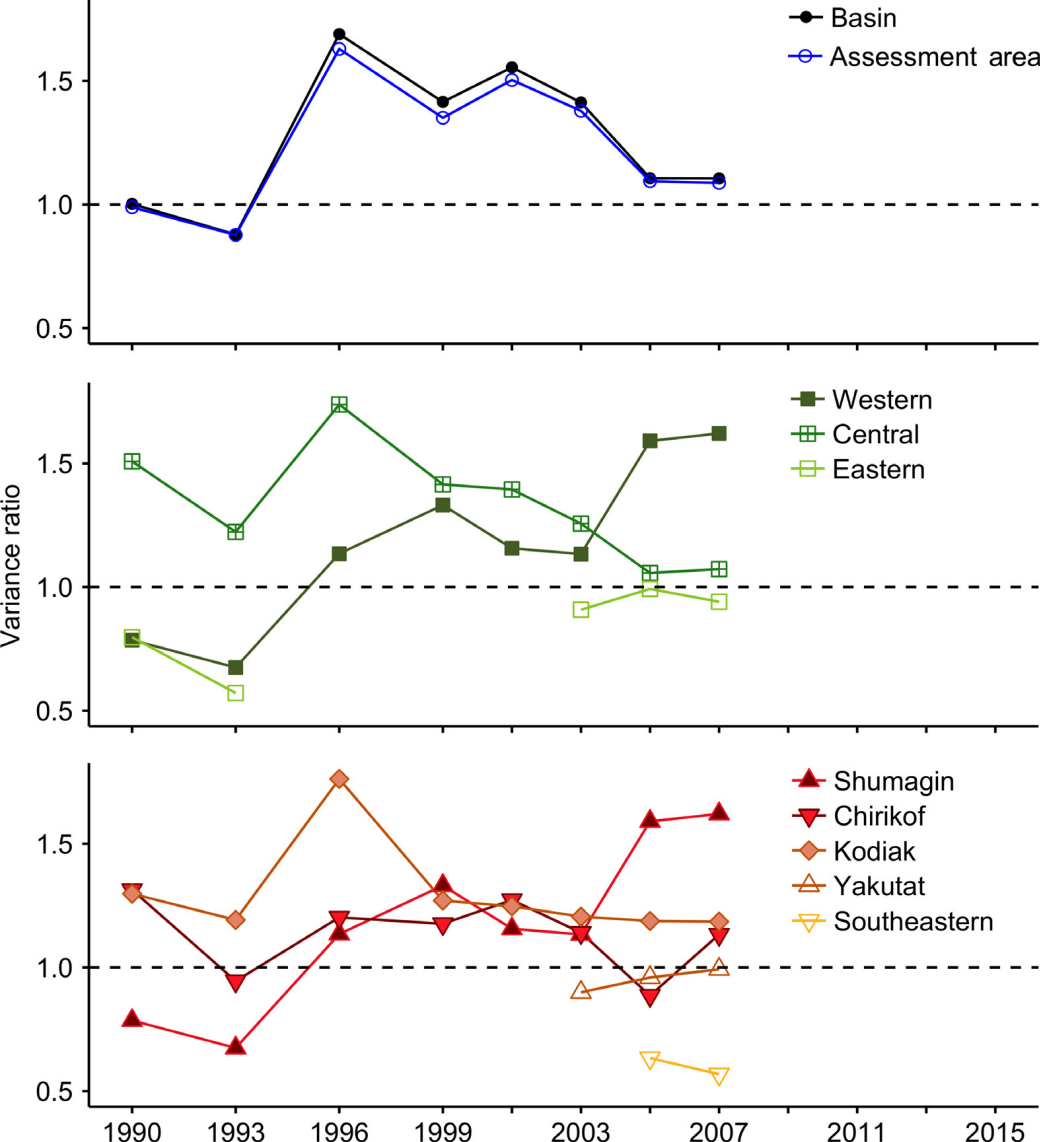
Variance ratios (VR*_j_*), by area and survey year. Each point denotes a variance ratio calculated from five consecutive survey years of data, beginning with the corresponding year on the *x*‐axis (e.g., the point aligned with 1990 illustrates a variance ratio from consumption data in 1990, 1993, 1996, 1999, and 2001). Insufficient data (i.e., less than five consecutive survey years) precluded variance ratio calculations beyond 2007. VR_j_ > 1 (dashed line) depicts synchrony in Pollock consumption among groundfish predators., VR_j_ ≈ 1 (± 0.1) illustrates independent trends in consumption, and VR_j_ < 1 reflects asynchronous predator dynamics.

Variance ratios generally indicated synchronous trends in consumption among Pollock predators (VR*_i_*_,_ *_j_* > 1) and, thus, a lack of portfolio effects at all spatial scales (Fig. 7). The primary exception was the eastern Gulf of Alaska (i.e., Yakutat and Southeastern statistical areas), which showed asynchronous or independent consumption dynamics during the few survey years analyzed. At the basin and Pollock assessment area scales, consumption was independent (VR*_i_*_,_ *_j_* ≈ 1) or asynchronous (VR*_i_*_,_ *_j_* < 1) at start of the time series (i.e., 1990 and 1993), but shifted to more synchronous (VR*_i_*_,_ *_j_* > 1) dynamics thereafter (Fig. 7). This trend toward greater synchrony was most pronounced in the western subregion (i.e., Shumagin statistical area). Conversely, the degree of synchrony decreased in the central subregion (i.e., Chirikof and Kodiak statistical areas).

We found a positive correlation in Pollock consumption between Arrowtooth Flounder and Sablefish in the Kodiak statistical area and between Arrowtooth Flounder and Walleye Pollock in the Yakutat statistical area (Table 4). There were no other correlations between Arrowtooth Flounder and other predators at any other spatial scale. Positive correlations were common among predator species other than Arrowtooth Flounder, except in the Shumagin (i.e., western subregion) and Southeastern statistical areas, where no correlations were detected.

**Table 4:** Correlation coefficients for predator‐ and area‐specific consumption of Pollock in the Gulf of Alaska, 1990 to 2015.

	ATF	PC	PH	SBL	WEP
ATF				0.56_kod_*	0.82_yak_*
PC			0.62_b_** 0.62_a_** 0.83_c_*** 0.79_chir_** 0.93_kod_***	0.98_e_*** 0.98_yak_***	0.82_c_** 0.81_yak_*
PH				0.63_b_** 0.63_a_** 0.59_chir_**	0.57_c_*
SBL					
WEP					

Notes

ATF, Arrowtooth Flounder; PC, Pacific Cod; PH, Pacific Halibut; SBL, Sablefish; WEP, Walleye Pollock. Only significant correlations (*P < 0.1, **P < 0.05, ***P < 0.001) are shown for each area (b, basin; a, area encompassed by the stock assessment for Gulf of Alaska Pollock; w, western Gulf of Alaska [i.e., Shumagin statistical area]; c, central Gulf of Alaska; e, eastern Gulf of Alaska; chir, Chirikof statistical area; kod, Kodiak statistical area; yak, Yakutat statistical area; and se, Southeastern statistical area).

## Discussion

We found Arrowtooth Flounder to be the dominant Pollock predator in the Gulf of Alaska, regardless of survey year or spatial scale. Although remaining groundfish predators consumed much less Pollock, trends in predation were synchronous among them. The combination of a single dominant predator and synchronous consumption dynamics indicates potential for strong top‐down control over Pollock in the Gulf of Alaska. We also found basin‐scale shifts from asynchronous or independent predator dynamics to more synchronized consumption, suggesting diminished trophic stability through time. Temporal trends in the degree of synchrony, however, varied by scale and individual location. This reflects substantial heterogeneity that may buffer against greater trophic instability in the region. In particular, opposing trends in synchrony between the western and central subregions may have helped preserve food web structure and function throughout the Gulf of Alaska in recent years.

### Variation in predation intensity

Although Walleye Pollock represents an important prey source for many economically important species, we found that consumption was not uniformly distributed among groundfishes in the Gulf of Alaska. Arrowtooth Flounder was the dominant Pollock predator, representing 74% of total consumption (by the five species examined). Food web models parameterized using the same bottom trawl survey and food habits data estimated that 54.5% of Pollock predation mortality was due to consumption by Arrowtooth Flounder, 12.1% by Pacific Cod, 15.4% by Pacific Halibut, 4.2% by Sablefish, and 13.8% by Walleye Pollock (Aydin et al. 2007, Gaichas et al. 2010). We attribute species‐specific differences in predation mortality between our study and previous research to the different time periods analyzed. The food webs models constructed by Aydin et al. (2007) and Gaichas et al. (2010) relied on three survey years of data (1990, 1993, and 1996), whereas we analyzed bottom trawl survey and diet data from 1990 to 2015 (i.e., 12 survey years of data). The latter portion of the time series showed a decline in total Pollock consumption that was more pronounced for Pacific Cod, Pacific Halibut, Sablefish, and Walleye Pollock, thereby increasing the relative contributions of Arrowtooth Flounder to Pollock predation mortality. When we calculated predation indices using only the first few years of the time series (i.e., 1990, 1993, and 1996; the same survey years used in regional food web models), predator‐specific contributions to Pollock mortality were more similar (i.e., 59% Arrowtooth Flounder, 25% Pacific Halibut, 8% Pacific Cod, 6% Sablefish, and 2% Walleye Pollock). However, absolute predation intensity was much greater in our study. For example, Hollowed et al. (2000b) estimated predation mortality by Arrowtooth Flounder to be 3.0 × 10^5^ Tg in 1997. Another study by van Kirk et al. (2010) estimated consumption by Arrowtooth Flounder to be 1.7 × 10^5^ Tg that same year. Our estimate for 1997 (a mean of consumption in 1996 and 1999) was considerably higher, at 3.8 × 10^6^ Tg of Pollock. Because of these disparities, we recommend that predation indices be used to track relative changes rather than infer absolute removals by groundfish predators.

Throughout much of the time series, consumption was greater than assessment‐based estimates of total Pollock biomass. Food web models have also indicated periods when predation mortality exceeded production (Dorn et al. 2017). However, there are several potential reasons why our estimates of predation intensity may have differed from those provided by other authors. First, our values for ${\bar{C}}_{s,i,j}$ were likely biased high because they were calculated from bioenergetics models that assumed predators fed at their theoretical maximum consumption rates (*C*_max_ = 1). Although theoretical maximum consumption may be appropriate for Arrowtooth Flounder (*C*_≥40 cm_ = 1.07), most fishes feed at rates less than half their *C*_max_ (i.e., median proportion of *C*_max_ = 0.43 across 66 populations from 38 species; Armstrong and Schindler 2011). Relative foraging rates are available for all of our focal species (Appendix S1: Table S5; Harvey 2009, Holsman and Aydin 2015) and could be used to modify *C*_max_ when calculating ${\bar{C}}_{s,i,j}$ (sensu Holsman and Aydin 2015, Spencer et al. 2016). Additionally, we calculated *C*_max_ using bottom temperatures from summer surveys. Effective foraging days, which make use of the von Bertalanffy growth function to integrate physical and trophodynamic processes over the course of a year, allowed for scaling from daily consumption to annual rations (Holsman and Aydin 2015). However, we did not directly account for cooler temperatures (and thus, decreased metabolic rates) in fall, winter, or spring. We also relied upon empirical data from European plaice (*Pleuronectes platessa;* Fonds et al. 1992) to estimate bioenergetics parameters for Arrowtooth Flounder (Holsman and Aydin 2015). This is because laboratory experiments aimed at parameterizing allometric consumption and temperature scaling functions for Arrowtooth Flounder have been unsuccessful due to a lack of foraging in captivity (K. Holsman, *personal observation*). With Arrowtooth Flounder being identified as the dominant Pollock predator, species‐specific bioenergetics parameters would improve predation indices as well as enhance our understanding about trophic stability in the Gulf of Alaska.

Another methodological explanation for differences between our results and prior estimates of Pollock predation is that we weighted food habits data to correct for sampling biases. Fork length and biomass weighting are not consistently incorporated into dietary analyses for groundfish predators in the Gulf of Alaska. Aydin et al. (2007) and Gaichas et al. (2010) biomass‐weighted food habits data to account for spatial differences in multispecies models. Given that diet data were obtained from a size‐structured sampling design, weighting by predator fork length is also necessary to scale to population levels (sensu Livingston et al. 2017). In most cases, we found that weighting diet data by fork length did not drastically alter *p_s_*_,_ *_i_*_,_ *_j_* (Appendix S1: Fig. S5). Predation indices were sensitive to proportional diet data, however, with small variations being magnified by other components (e.g.,* B_s_*_,_ *_i_*).

Finally, our predation index was designed to represent consumption by assessed groundfish predators (i.e., Arrowtooth Flounder ≥ 19 cm, Pacific Cod ≥ 0 cm, Pacific Halibut ≥ 82 cm, Sablefish ≥ 45 cm, and Walleye Pollock ≥ 37 cm). In doing so, we emphasized the size classes most likely to consume Pollock. Including smaller size classes and other predators (e.g., Steller sea lions [*Eumetopias jubatus*]) would increase absolute estimates of Pollock predation, though spatiotemporal trends should be robust because we have already accounted for all major sources of Pollock predation (Dorn et al. 2017).

### Predator dominance

Arrowtooth Flounder comprise the greatest biomass of any tertiary consumer in the Gulf of Alaska (Spies et al. 2017). They also maintain an extensive network of food web connections (Gaichas and Francis 2008). As a result, regional ecosystem models indicate that minor changes in Arrowtooth Flounder abundance can have considerable impacts on a variety of interacting species (Aydin et al. 2007). We found that Arrowtooth Flounder biomass (*B_s_*_,_ *_i_*) and their relative contributions to Pollock predation mortality (*P_a_*_,_ *_i_*_,_ *_j_*) followed the same general trends, increasing from 1993 to 2007 and decreasing thereafter. Although a recent multispecies model found a small negative correlation between Arrowtooth Flounder and Walleye Pollock (Thorson et al. 2019), opposite *B_s_*_,_ *_i_* trajectories and a predator assemblage that is dominated by Arrowtooth Flounder support the hypothesis for strong top‐down control over Gulf of Alaska Pollock (Hollowed et al. 2000b, Aydin et al. 2007, Gaichas et al. 2010, van Kirk et al. 2010, Holsman et al. 2016). Oken et al. (2018) reached a similar conclusion regarding the effects of Arrowtooth Flounder on Pacific herring (*Clupea pallasii*), suggesting that their predatory control is not limited to Pollock.

### Synchrony and trophic stability

Asynchronous dynamics among species or locations may generate portfolio effects (McNaughton 1977, Hooper et al. 2005, Schindler et al. 2015) that help buffer against strong predatory control. Variance ratios from our study generally reflected synchronous trends in Pollock consumption, a lack of portfolio effects, and potential trophic instability at the basin scale. These findings support previous claims of low predictability and high potential for predatory control in the “top heavy” Gulf of Alaska food web (Gaichas et al. 2015). The central subregion, however, showed a decrease in synchrony through time, suggesting increased trophic stability despite considerable predation pressure. Additionally, asynchronous and/or independent consumption dynamics were evident in the Yakutat and Southeastern statistical areas. The existence of different trajectories of two or more adjacent areas, in terms of synchrony and portfolio effects, supports the notion that spatial heterogeneity promotes community stability (Schindler et al. 2010). In fact, spatial asynchrony can generate greater portfolio effects than asynchronous trends among species (Thorson et al. 2018). This is an important consideration in the context of diversity–stability relationships in systems comprised of few dominant predators (e.g., Baltic Sea sprat, Eastern Scotian forage fishes, and Pacific herring in the Gulf of Alaska; Oken et al. 2018).

Continued data collection, especially in the eastern subregion, would strengthen our understanding about trophodynamics in the Gulf of Alaska and portfolio effects in a food web context. A longer time series would also help identify bottom‐up and/or top‐down mechanisms for trophic (in)stability in large marine ecosystems that regularly undergo shifts in community composition. Modeling predation intensity as a function of key environmental variables (sensu Litzow and Ciannelli 2007) would also contribute to our understanding about reorganization within the demersal fish community, especially in response to rapid climate change.

### Implications for fisheries management

We found that total Pollock consumption (*P_a_*_,_ *_i_*_,_ *_j_*) exceeded assessment‐based estimates of Pollock biomass (*B_s_*_,_ *_i_*) in over half of the study period. There was also substantial variation in *P_a_*_,_ *_i_*_,_ *_j_* at all spatial scales, including the area encompassed by the stock assessment for Gulf of Alaska Pollock. High consumption‐to‐biomass ratios and considerable variation in predation intensity suggest that time‐varying estimates of natural mortality may benefit the assessment for Gulf of Alaska Pollock. Regional differences in predation intensity and community stability also promote a spatially explicit approach (e.g., Spencer et al. 2016). A plethora of case studies (e.g., Magnusson 1995, Gislason 1999, Hollowed et al. 2000a, Jurado‐Molina et al. 2005, Moustahfid et al. 2009, van Kirk et al. 2010, Tyrrell et al. 2011, Holsman et al. 2016) have shown that including ecological parameters such as predation directly into stock assessment models can impact the magnitude and uncertainty of biological reference points. To date, estimates of Pollock predation mortality in the Gulf of Alaska have relied on highly complex multispecies models. A simpler approach to operationalizing ecosystem‐based fisheries management would be to use changes in predation intensity as a modifier of assumed constant natural mortality (e.g., Hollowed et al. 2000b, Livingston and Methot 1998, A’mar et al. 2010, Spencer et al. 2016). We assert that our empirically derived, time‐varying, spatially explicit, and age‐structured predation index is well suited to account for complex ecological processes in this way. When direct incorporation into stock assessment models is not feasible, predation mortality can be included as part of an ecosystem and socioeconomic profile (ESP), a standardized appendix that provides relevant indicators to help inform stock status (e.g., Shotwell et al. 2019). Although our results are most applicable to the species, areas, and time periods included in this case study, our analytical approach can be used to estimate predation mortality and better understand trophic stability in large marine ecosystems around the world.

## Supporting information

Appendix S1

## Data Availability

Data sources and script files are available on Zenodo: https://doi.org/10.5281/zenodo.3723950
