# Supplementary material for: Development of a predation index to assess trophic stability in the Gulf of Alaska
Source: Ecol Appl. 2020 Jul 31;30(7):e02141. doi: 10.1002/eap.2141 (PMC7583375; doi:10.1002/eap.2141)
Supplement: Supplementary file 1 — Appendix S1 [file EAP-30-e02141-s001.pdf]

Supporting Information. Barnes, C.L., A.H. Beaudreau, M.W. Dorn, K.H. Holsman, and F.J. Mueter. 2020. Development of a predation index to assess trophic stability in the Gulf of Alaska. Ecological Applications.

Appendix S1

Standardized Survey Designs

The Alaska Fisheries Science Center (AFSC, National Oceanic and Atmospheric Administration) used a stratified random sampling design to conduct surveys along the continental shelf, triennially from 1990 to 1999 and biennially from 2001 onward (von Szalay and Raring 2015; data publicly available at https://www.afsc.noaa.gov/RACE/groundfish/survey_data/data.htm). The Yakutat and Southeastern statistical areas were not surveyed in 2001. Date, location (latitude and longitude), gear depth (m), and bottom temperature (°C) were recorded for each tow. Fishes caught were identified to species and weighed for calculations of catch-per-unit-effort (CPUE; kg per hectare). Fork lengths were measured from a random subsample of 100 to 300 fish per species and haul. Walleye Pollock and a variety of other species were randomly sampled for ageing.

The International Pacific Halibut Commission (IPHC) started conducting annual setline surveys targeting Pacific Halibut in 1998 (Clark and Hare 2006; data publicly available at https://iphc.int/data/fiss-data-query). Individual setline stations (delineated as 10 nm^2 grid cells) were systematically sampled across the continental shelf, with setline skates deployed in all Gulf of Alaska regulatory areas (*i.e.*, 4A, 3B, 3A, and 2C) throughout the summer. IPHC staff recorded date, latitude, longitude, and gear depth (fm) at each station. Fork lengths (in) were measured and weights (lb) were calculated using known length-weight relationships. CPUE was estimated as the total weight (lb) of halibut caught per effective skate (*i.e.*, 100 standardized

circle hooks with 18 ft spacing). For the purposes of this study, we converted depths, fish lengths, and estimated weights from imperial to metric units (m, cm, g).

The AFSC's Auke Bay Laboratories and Resource Assessment and Conservation Engineering (RACE Division) jointly administered longline surveys targeting Sablefish and other groundfishes annually since 1979 (Sigler and Zenger 1989; data publicly available at https://www.afsc.noaa.gov/maps/longline/Map.php). Systematic surveys took place in select gullies along the upper continental shelf in summer, beginning in the western Gulf of Alaska and ending in the eastern Gulf of Alaska. Date, latitude, longitude, gear depth (m), and the number of effective skates (*i.e.*, 100 m line with 45 baited circle hooks spaced at 2 m) were recorded at each station. Fork lengths (mm) were measured and weights (kg) were calculated from known length-weight relationships.

Food Habits Data Collection

The AFSC subsampled up to five stomachs per species, haul, and size class between 1990 and 2015. The Yakutat statistical area was not subsampled in 1996, 1999, or 2001 and the Southeastern statistical area was not subsampled prior to 2005. Additionally, Sablefish were not subsampled in 2013 or 2015. Size classes targeted for stomach content analyses by the Resource Ecology and Ecosystem Modeling (REEM) program were a) < 31 cm, 31 to 50 cm, 51 to 70 cm, and > 70 cm for Arrowtooth Flounder, Pacific Cod, and Pacific Halibut, b) < 50 cm, 50 to 59 cm, 60 to 69 cm, and ≥ 70 cm for Sablefish, and c) < 30 cm, 30 to 39 cm, 40 to 49 cm, and ≥ 50 cm for Walleye Pollock (Hibpshman *et al.* 2017). Prey from non-empty stomachs were identified to the lowest possible taxonomic group and weighed (0.001 g; Livingston *et al.* 2017). REEM personnel measured standard lengths (mm) from fish prey, including Walleye Pollock, whenever

possible. All food habits data used to calculate proportions of pollock consumed can be found at: https://access.afsc.noaa.gov/REEM/WebDietData/DietDataIntro.php.

Table S1. Parameters α and β used to estimate weight from length for Arrowtooth Flounder (ATF), Pacific Cod (PC), Sablefish (SBL; f: female, m: male), and Walleye Pollock (WEP). The bias-correction factor for Walleye Pollock and references for species-specific relationships are also shown. Individual weights for Pacific Halibut were provided in the International Pacific Halibut Commission's setline survey database.

Species	α	β	cf	Reference
ATF	4.312×10^{-3}	3.1860	-	Spies *et al.* 2017
PC	5.631×10^{-6}	3.1306	-	Barbeaux *et al.* 2017
PH	-	-	-	-
SBL_f	1.010×10^{-5}	3.0150	-	Hanselman *et al.* 2007
SBL_m	1.240×10^{-5}	2.9600	-	Hanselman *et al.* 2007
WEP	5.616×10^{-6}	3.0447	1.007	Dorn *et al.* 2017; Brodziak 2012

Table S2. Results for the top three generalized additive models (without spatial autocorrelation) used to quantify presence-absence and log-CPUE of positive catches, by species. X indicates variables (survey year, longitude and latitude, depth [m], bottom temperature [°C]) included in each alternative model. The deviance explained (Dev. %), equivalent degrees of freedom (edf), log-likelihood (LL), ΔAIC, Akaike weight (W_i), and unbiased risk estimator (UBRE) are noted. Selected models are shown in bold.

Model	Variables Included				Dev. (%)	edf	LL	ΔAIC	W_i	GCV / UBRE
	Year	Lon, Lat	Depth	Temp						
**Arrowtooth Flounder**										
Presence-absence	**X**	**X**	**X**	**X**	**44.9**	**49**	**- 1534**	**0.0**	**0.998**	**- 0.633**
	X	X	X		44.6	48	- 1541	12.7	0.002	- 0.632
		X	X	X	43.8	37	- 1566	40.3	0.000	- 0.629
CPUE, where present	**X**	**X**	**X**	**X**	**36.0**	**52**	**- 13869**	**0.0**	**1.000**	**2.095**
	X	X	X		35.8	50	- 13881	18.6	0.000	2.100
		X	X	X	34.8	40	- 13937	112.9	0.000	2.125
**Pacific Cod**										
Presence-absence	**X**	**X**	**X**	**X**	**29.8**	**45**	**- 4007**	**0.0**	**1.000**	**- 0.062**
	X	X	X		29.5	42	- 4020	22.2	0.000	- 0.059
		X	X	X	27.2	33	- 4152	267.0	0.000	- 0.031
CPUE, where present	**X**	**X**	**X**	**X**	**11.0**	**47**	**- 10121**	**0.0**	**1.000**	**2.506**
	X	X	X		10.4	47	- 10137	32.3	0.000	2.521
	X	X		X	9.4	41	- 10167	81.1	0.000	2.544

Table S2 (cont). Results for the top three generalized additive models (without spatial autocorrelation) used to quantify presence-absence and log-CPUE of positive catches, by species. X indicates variables (survey year, longitude and latitude, depth [m], bottom temperature [°C]) included in each alternative model. The deviance explained (Dev. %), equivalent degrees of freedom (df), log-likelihood (LL), ΔAIC, Akaike weight (W_i), and unbiased risk estimator (UBRE) are noted. Selected models are shown in bold.

	Variables Included in Alt. Model									
Model	Year	Lon, Lat	Depth	Temp	Dev. (%)	edf	LL	ΔAIC	W_i	GCV / UBRE
**Pacific Halibut**										
Presence-absence	**X**	**X**	**X**	**-**	**36.8**	**54**	**- 924**	**0.0**	**1.000**	**- 0.875**
		X	X	-	35.0	34	- 952	16.5	0.000	- 0.874
	X	X		-	31.1	48	- 1008	155.6	0.000	- 0.865
CPUE, where present	**X**	**X**	**X**	**-**	**32.0**	**56**	**- 21128**	**0.0**	**1.000**	**0.925**
	X	X		-	30.7	48	- 21275	278.5	0.000	0.942
	X		X	-	17.5	25	- 22615	2912.2	0.000	1.119
**Sablefish**										
Presence-absence	-	-	-	-	-	-	-	-	-	-
	-	-	-	-	-	-	-	-	-	-
	-	-	-	-	-	-	-	-	-	-
CPUE, where present	**X**	**X**	**X**	**-**	**63.2**	**62**	**- 1762**	**0.00**	**1.000**	**0.343**
		X	X	-	54.1	34	- 1991	401.9	0.000	0.417
	X	X		-	53.6	57	- 2002	469.8	0.000	0.431

Table S2 (cont). Results for the top three generalized additive models (without spatial autocorrelation) used to quantify presence-absence and log-CPUE of positive catches, by species. X indicates variables (survey year, longitude and latitude, depth [m], bottom temperature [°C]) included in each alternative model. The deviance explained (Dev. %), equivalent degrees of freedom (edf), log-likelihood (LL), ΔAIC, Akaike weight (W_i), and unbiased risk estimator (UBRE) are noted. Selected models are shown in bold.

	Variables Included in Alt. Model									
Model	Year	Lon, Lat	Depth	Temp	Dev. (%)	edf	LL	**Δ**AIC	W_i	GCV / UBRE
**Walleye Pollock**										
Presence-absence	**X**	**X**	**X**	**X**	**28.3**	**52**	**- 4244**	**0.0**	**1.000**	**- 0.005**
	X	X	X		28.1	49	- 4257	21.7	0.000	- 0.003
		X	X	X	25.6	39	- 4406	298.8	0.000	0.029
CPUE, where present	**X**	**X**	**X**	**X**	**15.3**	**50**	**- 10616**	**0.0**	**0.971**	**4.744**
	X	X	X		15.1	47	- 10622	7.0	0.029	4.751
		X	X	X	12.7	38	- 10690	124.7	0.000	4.867

Table S3. Akaike information criterion (expressed as ΔAIC) for generalized additive mixed models (GAMM) with and without a Gaussian spatial autocorrelation term for Arrowtooth Flounder (ATF), Pacific Cod (PC), Pacific Halibut (PH), Sablefish (SBL), and Walleye Pollock (WEP). Equivalent degrees of freedom (rounded to the nearest whole number) are shown in parentheses. Selected models are shown in bold. GAMMs would not converge when modeling presence-absence of Pacific Halibut. Sablefish were observed at nearly all stations, thus eliminating the need to model presence-absence for this species.

	Presence-Absence		log-CPUE	
Model	GAMM w/o	GAMM w/	GAMM w/o	GAMM w/
ATF	317 (48)	**0 (49)**	25 (52)	**0 (52)**
PC	144 (43)	**0 (47)**	7 (42)	**0 (42)**
PH	**-**	-	980 (54)	**0 (55)**
SBL	N/A	N/A	**0 (60)**	2 (60)
WEP	**0 (51)**	7 (51)	37 (48)	**0 (48)**

Table S4. Parameter estimates from best-fit models used to quantify presence-absence and CPUE for a) Arrowtooth Flounder, b) Pacific Cod, c) Pacific Halibut, d) Sablefish, and e) Walleye Pollock in the Gulf of Alaska. Year was treated as a factor. Thus, 1990 is denoted as the model intercept except in the case of Pacific Halibut, where the intercept represents 1998. Subsequent estimates are shown as differences from the model intercept. Although there were additional survey years for Pacific Halibut and Sablefish, only those that coincided with the bottom trawl survey are shown. Non-significant terms (α = 0.1) are in gray.

a) Arrowtooth Flounder

Model	Estimate	Std. Error	z- or t-value	edf	Chi Sq. or F	p-value	Adj. R^2
Presence-Absence							0.408
(intercept)	3.35	0.32	10.58			< 0.001	
1993	0.44	0.35	1.24			0.214	
1996	0.15	0.35	0.45			0.656	
1999	0.66	0.36	1.85			0.065	
2001	0.46	0.36	1.26			0.208	
2003	0.84	0.35	2.42			0.015	
2005	1.32	0.35	3.74			< 0.001	
2007	0.43	0.35	1.22			0.221	
2009	1.18	0.36	3.26			0.001	
2011	1.28	0.36	3.52			< 0.001	
2013	0.64	0.36	1.77			0.077	
2015	0.91	0.35	2.58			0.010	
2017	1.22	0.38	3.25			0.001	
Lon, Lat				28.23	371.75	< 0.001	
Depth				7.17	759.06	< 0.001	
Bottom Temp				1.08	15.34	< 0.001	

Table S4 (cont). Parameter estimates from best-fit models used to quantify presence-absence and CPUE for a) Arrowtooth Flounder, b) Pacific Cod, c) Pacific Halibut, d) Sablefish, and e) Walleye Pollock in the Gulf of Alaska. Year was treated as a factor. Thus, 1990 is denoted as the model intercept except in the case of Pacific Halibut, where the intercept represents 1998. Subsequent estimates are shown as differences from the model intercept. Although there were additional survey years for Pacific Halibut and Sablefish, only those that coincided with the bottom trawl survey are shown. Non-significant terms (α = 0.1) are in gray.

a) Arrowtooth Flounder

Model	Estimate	Std. Error	z- or t-value	edf	Chi Sq. or F	p-value	Adj. R^2
CPUE, kg per ha							0.355
(intercept)	7.10	0.09	78.50			< 0.001	
1993	0.38	0.11	3.63			< 0.001	
1996	0.22	0.11	2.07			0.039	
1999	0.31	0.11	2.86			0.004	
2001	0.27	0.12	2.30			0.022	
2003	0.59	0.11	5.49			< 0.001	
2005	0.65	0.11	6.15			< 0.001	
2007	0.41	0.11	3.77			< 0.001	
2009	0.45	0.11	4.20			< 0.001	
2011	0.38	0.11	3.46			< 0.001	
2013	0.06	0.11	0.49			0.622	
2015	0.25	0.11	2.32			0.020	
2017	- 0.05	0.11	- 0.42			0.677	
Lon, Lat				28.37	55.82	< 0.001	
Depth				8.02	239.55	< 0.001	
Bottom Temp				2.53	8.20	< 0.001	

Table S4 (cont). Parameter estimates from best-fit models used to quantify presence-absence and CPUE for a) Arrowtooth Flounder, b) Pacific Cod, c) Pacific Halibut, d) Sablefish, and e) Walleye Pollock in the Gulf of Alaska. Year was treated as a factor. Thus, 1990 is denoted as the model intercept except in the case of Pacific Halibut, where the intercept represents 1998. Subsequent estimates are shown as differences from the model intercept. Although there were additional survey years for Pacific Halibut and Sablefish, only those that coincided with the bottom trawl survey are shown. Non-significant terms (α = 0.1) are in gray.

b) Pacific Cod

Model	Estimate	Std. Error	z- or t-value	edf	Chi Sq. or F	p-value	Adj. R^2
Presence-Absence							0.348
(intercept)	0.96	0.50	1.90			0.058	
1993	- 0.34	0.19	- 1.80			0.072	
1996	- 0.84	0.19	- 4.45			< 0.001	
1999	- 1.27	0.19	- 6.69			< 0.001	
2001	- 1.90	0.20	- 9.64			< 0.001	
2003	- 1.44	0.19	- 7.78			< 0.001	
2005	- 1.46	0.18	- 7.93			< 0.001	
2007	- 1.57	0.19	- 8.22			< 0.001	
2009	- 0.97	0.19	- 5.05			< 0.001	
2011	- 0.87	0.19	- 4.50			< 0.001	
2013	- 0.99	0.20	- 4.98			< 0.001	
2015	- 0.82	0.19	- 4.31			< 0.001	
2017	- 1.91	0.19	- 9.87			< 0.001	
Lon, Lat				25.67	821.80	< 0.001	
Depth				4.58	552.30	< 0.001	
Bottom Temp				2.07	25.50	< 0.001	

Table S4 (cont). Parameter estimates from best-fit models used to quantify presence-absence and CPUE for a) Arrowtooth Flounder, b) Pacific Cod, c) Pacific Halibut, d) Sablefish, and e) Walleye Pollock in the Gulf of Alaska. Year was treated as a factor. Thus, 1990 is denoted as the model intercept except in the case of Pacific Halibut, where the intercept represents 1998. Subsequent estimates are shown as differences from the model intercept. Although there were additional survey years for Pacific Halibut and Sablefish, only those that coincided with the bottom trawl survey are shown. Non-significant terms (α = 0.1) are in gray.

b) Pacific Cod

Model	Estimate	Std. Error	z- or t-value	edf	Chi Sq. or F	p-value	Adj. R^2
CPUE, kg per ha							0.102
(intercept)	6.44	0.13	51.12			< 0.001	
1993	0.18	0.14	1.27			0.206	
1996	0.10	0.15	0.67			0.504	
1999	- 0.28	0.15	- 1.84			0.066	
2001	- 0.49	0.16	- 3.01			0.003	
2003	- 0.21	0.15	- 1.39			0.163	
2005	- 0.28	0.15	- 1.88			0.060	
2007	- 0.59	0.15	- 3.93			< 0.001	
2009	- 0.03	0.15	- 0.20			0.840	
2011	0.02	0.15	0.12			0.906	
2013	0.10	0.15	0.65			0.514	
2015	- 0.09	0.15	- 0.63			0.531	
2017	- 0.67	0.16	- 4.17			< 0.001	
Lon, Lat				25.20	6.99	< 0.001	
Depth				7.08	11.81	< 0.001	
Bottom Temp				1.37	25.51	< 0.001	

Table S4 (cont). Parameter estimates from best-fit models used to quantify presence-absence and CPUE for a) Arrowtooth Flounder, b) Pacific Cod, c) Pacific Halibut, d) Sablefish, and e) Walleye Pollock in the Gulf of Alaska. Year was treated as a factor. Thus, 1990 is denoted as the model intercept except in the case of Pacific Halibut, where the intercept represents 1998. Subsequent estimates are shown as differences from the model intercept. Although there were additional survey years for Pacific Halibut and Sablefish, only those that coincided with the bottom trawl survey are shown. Non-significant terms (α = 0.1) are in gray.

c) Pacific Halibut

Model	Estimate	Std. Error	z- or t-value	edf	Chi Sq. or F	p-value	Adj. R^2
Presence-Absence							0.218
(intercept *1998)	6.74	0.45	15.00			< 0.001	
1993	-	-	-			-	
1996	-	-	-			-	
1999	- 0.04	0.56	- 0.08			0.939	
2001	- 0.76	0.50	- 1.52			0.130	
2003	- 0.87	0.49	- 1.78			0.075	
2005	- 0.58	0.51	- 1.13			0.257	
2007	- 1.43	0.46	- 3.07			0.002	
2009	- 0.33	0.53	- 0.63			0.530	
2011	- 0.95	0.49	- 1.95			0.051	
2013	- 1.38	0.46	- 2.97			0.003	
2015	- 1.50	0.46	- 3.26			0.001	
2017	- 1.58	0.46	- 3.47			< 0.001	
Lon, Lat				26.75	439.2	< 0.001	
Depth				7.06	152.1	< 0.001	
Bottom Temp				-	-	-	

Table S4 (cont). Parameter estimates from best-fit models used to quantify presence-absence and CPUE for a) Arrowtooth Flounder, b) Pacific Cod, c) Pacific Halibut, d) Sablefish, and e) Walleye Pollock in the Gulf of Alaska. Year was treated as a factor. Thus, 1990 is denoted as the model intercept except in the case of Pacific Halibut, where the intercept represents 1998. Subsequent estimates are shown as differences from the model intercept. Although there were additional survey years for Pacific Halibut and Sablefish, only those that coincided with the bottom trawl survey are shown. Non-significant terms (α = 0.1) are in gray.

c) Pacific Halibut

Model	Estimate	Std. Error	z- or t-value	edf	Chi Sq. or F	p-value	Adj. R^2
CPUE, kg per ha							0.318
(intercept *1998)	6.53	0.03	189.67			< 0.001	
1993	-	-	-			-	
1996	-	-	-			-	
1999	- 0.07	0.05	- 1.35			0.178	
2001	- 0.48	0.05	- 9.78			< 0.001	
2003	- 0.20	0.05	- 4.09			< 0.001	
2005	- 0.37	0.05	- 7.58			< 0.001	
2007	- 0.93	0.05	- 18.98			< 0.001	
2009	- 0.90	0.05	- 18.49			< 0.001	
2011	- 1.25	0.05	- 25.49			< 0.001	
2013	- 1.30	0.05	- 26.54			< 0.001	
2015	- 1.22	0.05	- 24.85			< 0.001	
2017	- 1.53	0.05	- 31.20			< 0.001	
Lon, Lat				27.96	111.91	< 0.001	
Depth				7.80	34.42	< 0.001	
Bottom Temp				-	-	-	

Table S4 (cont). Parameter estimates from best-fit models used to quantify presence-absence and CPUE for a) Arrowtooth Flounder, b) Pacific Cod, c) Pacific Halibut, d) Sablefish, and e) Walleye Pollock in the Gulf of Alaska. Year was treated as a factor. Thus, 1990 is denoted as the model intercept except in the case of Pacific Halibut, where the intercept represents 1998. Subsequent estimates are shown as differences from the model intercept. Although there were additional survey years for Pacific Halibut and Sablefish, only those that coincided with the bottom trawl survey are shown. Non-significant terms ($\alpha = 0.1$) are in gray.

d) Sablefish

Model	Estimate	Std. Error	z- or t-value	edf	Chi Sq. or F	p-value	Adj. R^2
Presence-Absence							-
(intercept)	-	-	-			-	
1993	-	-	-			-	
1996	-	-	-			-	
1999	-	-	-			-	
2001	-	-	-			-	
2003	-	-	-			-	
2005	-	-	-			-	
2007	-	-	-			-	
2009	-	-	-			-	
2011	-	-	-			-	
2013	-	-	-			-	
2015	-	-	-			-	
2017	-	-	-			-	
Lon, Lat				-	-	-	
Depth				-	-	-	
Bottom Temp				-	-	-	

Table S4 (cont). Parameter estimates from best-fit models used to quantify presence-absence and CPUE for a) Arrowtooth Flounder, b) Pacific Cod, c) Pacific Halibut, d) Sablefish, and e) Walleye Pollock in the Gulf of Alaska. Year was treated as a factor. Thus, 1990 is denoted as the model intercept except in the case of Pacific Halibut, where the intercept represents 1998. Subsequent estimates are shown as differences from the model intercept. Although there were additional survey years for Pacific Halibut and Sablefish, only those that coincided with the bottom trawl survey are shown. Non-significant terms ($\alpha = 0.1$) are in gray.

d) Sablefish

Model	Estimate	Std. Error	z- or t-value	edf	Chi Sq. or F	p-value	Adj. R^2
CPUE, kg per ha							0.621
(intercept)	8.16	0.07	125.15			< 0.001	
1993	- 0.02	0.09	- 0.26			0.798	
1996	- 0.16	0.09	- 1.69			0.091	
1999	- 0.19	0.09	- 2.02			0.044	
2001	- 0.16	0.09	- 1.66			0.098	
2003	- 0.38	0.09	- 4.02			< 0.001	
2005	- 0.48	0.09	- 5.13			< 0.001	
2007	- 0.46	0.09	- 4.85			< 0.001	
2009	- 0.45	0.09	- 4.83			< 0.001	
2011	- 0.36	0.09	- 3.84			< 0.001	
2013	- 0.95	0.09	- 10.11			< 0.001	
2015	- 0.97	0.09	- 10.26			< 0.001	
2017	- 0.63	0.09	- 6.62			< 0.001	
Lon, Lat				27.37	25.95	< 0.001	
Depth				6.11	78.02	< 0.001	
Bottom Temp				-	-	-	

Table S4 (cont). Parameter estimates from best-fit models used to quantify presence-absence and CPUE for a) Arrowtooth Flounder, b) Pacific Cod, c) Pacific Halibut, d) Sablefish, and e) Walleye Pollock in the Gulf of Alaska. Year was treated as a factor. Thus, 1990 is denoted as the model intercept except in the case of Pacific Halibut, where the intercept represents 1998. Subsequent estimates are shown as differences from the model intercept. Although there were additional survey years for Pacific Halibut and Sablefish, only those that coincided with the bottom trawl survey are shown. Non-significant terms (α = 0.1) are in gray.

e) Walleye Pollock

Model	Estimate	Std. Error	z- or t-value	edf	Chi Sq. or F	p-value	Adj. R^2
Presence-Absence							0.342
(intercept)	1.24	0.16	7.52			< 0.001	
1993	- 0.12	0.19	- 0.64			0.524	
1996	- 0.61	0.19	- 3.24			0.001	
1999	- 0.74	0.19	- 3.92			< 0.001	
2001	- 1.77	0.21	- 8.62			< 0.001	
2003	- 0.87	0.19	- 4.64			< 0.001	
2005	- 1.17	0.18	- 6.34			< 0.001	
2007	- 1.70	0.19	- 8.89			< 0.001	
2009	- 0.97	0.19	- 5.13			< 0.001	
2011	- 0.55	0.19	- 2.87			0.004	
2013	- 0.85	0.20	- 4.38			< 0.001	
2015	- 0.09	0.19	- 0.47			0.642	
2017	- 0.71	0.20	- 3.62			< 0.001	
Lon, Lat				28.17	636.94	< 0.001	
Depth				8.13	1372.48	< 0.001	
Bottom Temp				2.35	24.94	< 0.001	

Table S4 (cont). Parameter estimates from best-fit models used to quantify presence-absence and CPUE for a) Arrowtooth Flounder, b) Pacific Cod, c) Pacific Halibut, d) Sablefish, and e) Walleye Pollock in the Gulf of Alaska. Year was treated as a factor. Thus, 1990 is denoted as the model intercept except in the case of Pacific Halibut, where the intercept represents 1998. Subsequent estimates are shown as differences from the model intercept. Although there were additional survey years for Pacific Halibut and Sablefish, only those that coincided with the bottom trawl survey are shown. Non-significant terms (α = 0.1) are in gray.

e) Walleye Pollock

Model	Estimate	Std. Error	z- or t-value	edf	Chi Sq. or F	p-value	Adj. R^2
CPUE, kg per ha							0.144
(intercept)	6.05	0.15	39.71			< 0.001	
1993	- 0.62	0.18	- 3.48			< 0.001	
1996	- 0.66	0.18	- 3.58			< 0.001	
1999	- 0.67	0.19	- 3.53			< 0.001	
2001	- 0.99	0.23	- 4.33			< 0.001	
2003	- 0.58	0.19	- 3.08			0.002	
2005	- 0.31	0.19	- 1.63			0.103	
2007	- 0.54	0.20	- 2.68			0.007	
2009	0.14	0.19	0.74			0.458	
2011	- 0.09	0.19	- 0.47			0.639	
2013	0.11	0.20	0.57			0.570	
2015	- 0.28	0.18	- 1.52			0.128	
2017	- 1.36	0.20	- 6.91			< 0.001	
Lon, Lat				28.26	20.15	< 0.001	
Depth				5.92	24.01	< 0.001	
Bottom Temp				2.41	2.47	0.037	

Table S5. Relative foraging rates (RFR; proportion of C_{max}) for Arrowtooth Flounder, Pacific Cod, Pacific Halibut, Sablefish, and Walleye Pollock in the Gulf of Alaska. References for each size-specific estimate are also shown.

Predator	RFR	Reference
Arrowtooth Flounder	< 40 cm: 0.79<br>≥ 40 cm: 1.07	Holsman and Aydin 2015
Pacific Cod	< 55 cm: 0.41<br>≥ 55 cm: 0.47	Holsman and Aydin 2015
Pacific Halibut	< 40 cm: 0.26<br>40 – 120 cm: 0.40	Holsman *et al.* 2019
Sablefish	40 – 50 cm: 0.27<br>≥ 50 cm: 0.26	Harvey 2009
Walleye Pollock	< 40 cm: 0.49<br>≥ 40 cm: 0.56	Holsman and Aydin 2015

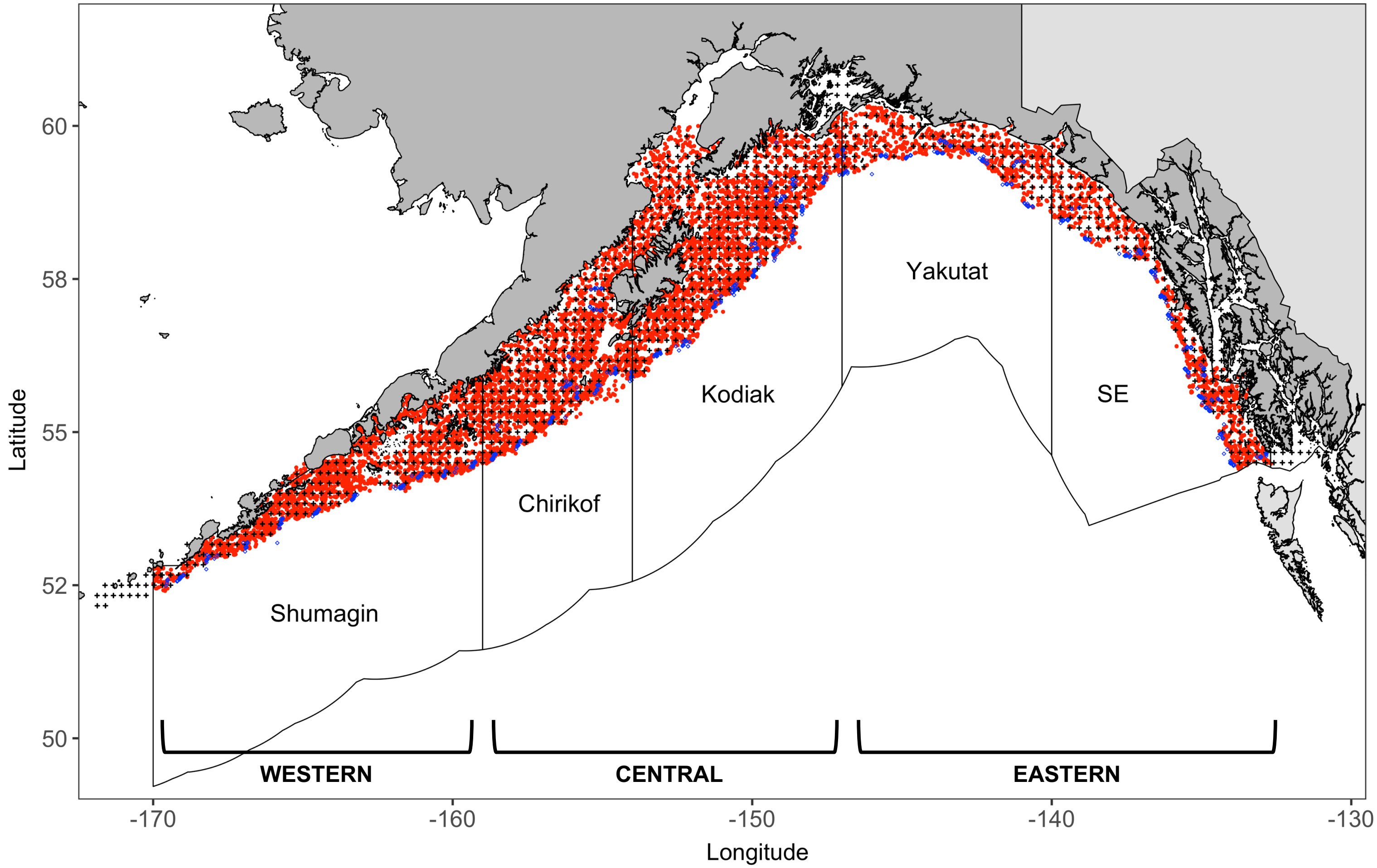

Figure S1. Map of study area (Gulf of Alaska, 1990 to 2015). Red dots illustrate tow locations for the Alaska Fisheries Science Center (AFSC) bottom trawl survey. Black crosses denote International Pacific Halibut Commission (IPHC) setline survey stations. Blue dots represent AFSC longline survey stations. Unfilled polygons denote statistical areas defined by the International North Pacific Fisheries Commission (INPFC). Subregions are shown in bold.

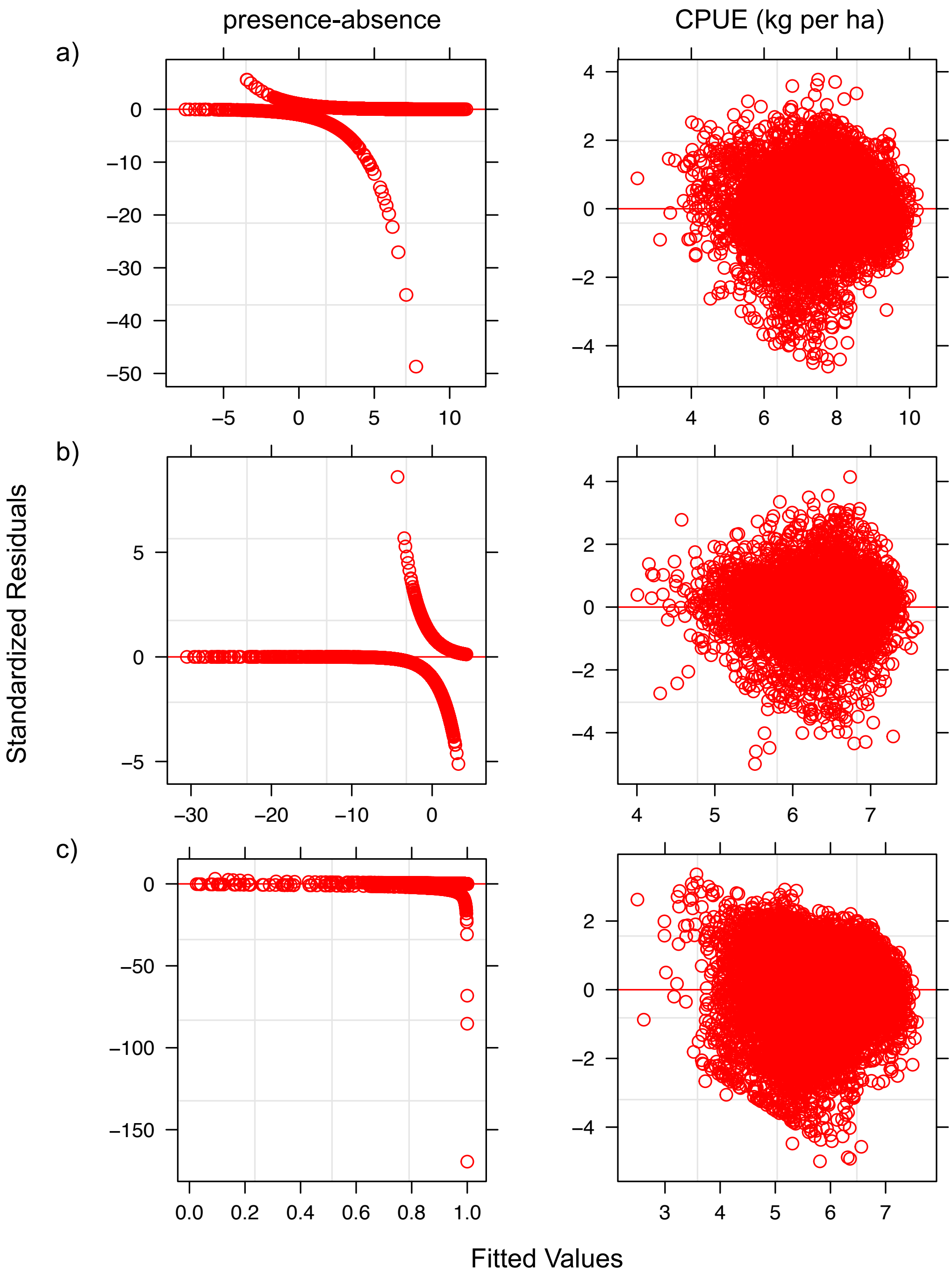

Figure S2. Residuals from best-fit models used to quantify presence-absence (left) and catch-per-unit-effort (CPUE; kg per ha) for a) Arrowtooth Flounder, b) Pacific Cod, c) Pacific Halibut, d) Sablefish, and e) Walleye Pollock (Gulf of Alaska). Model covariates included survey year (1990 to 2015), latitude and longitude, depth (m), and bottom temperature, if available (°C).

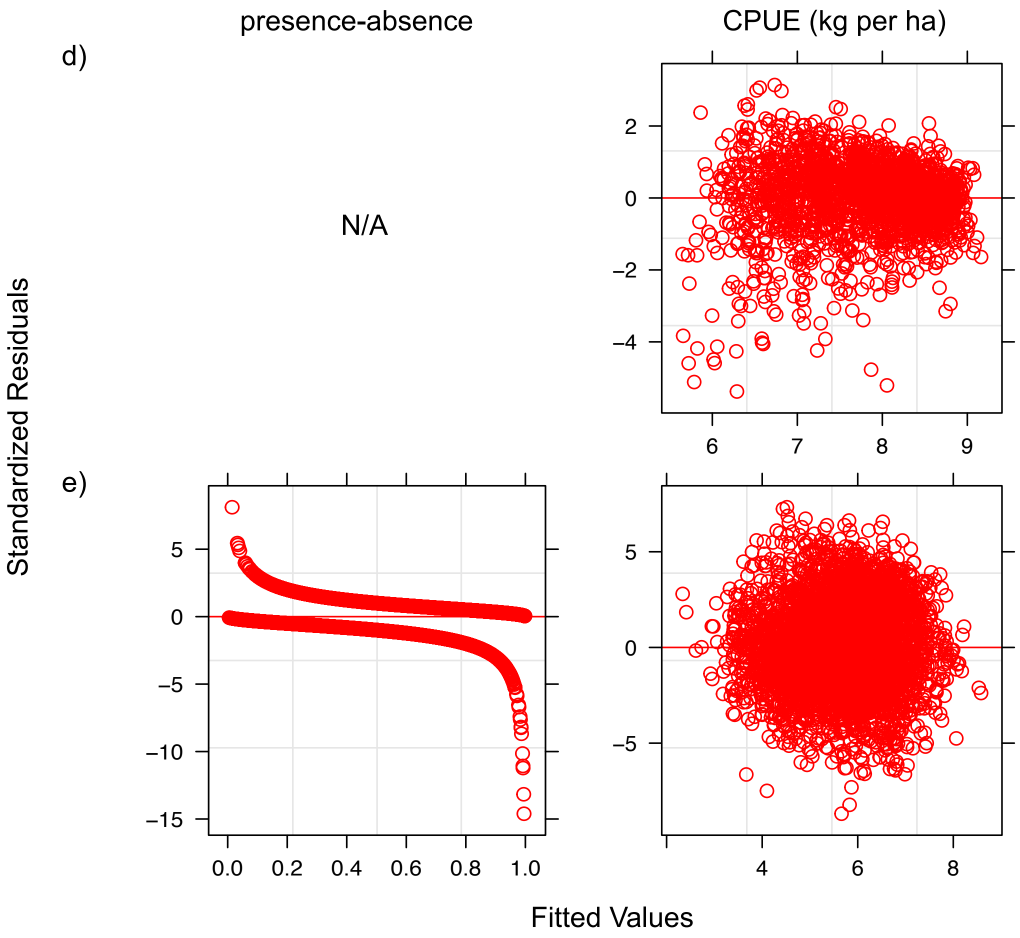

Figure S2 (cont). Residuals from best-fit models used to quantify presence-absence (left) and catch-per-unit-effort (CPUE; kg per ha) for a) Arrowtooth Flounder, b) Pacific Cod, c) Pacific Halibut, d) Sablefish, and e) Walleye Pollock (Gulf of Alaska). Model covariates included survey year (1990 to 2015), latitude and longitude, depth (m), and bottom temperature, if available (°C).

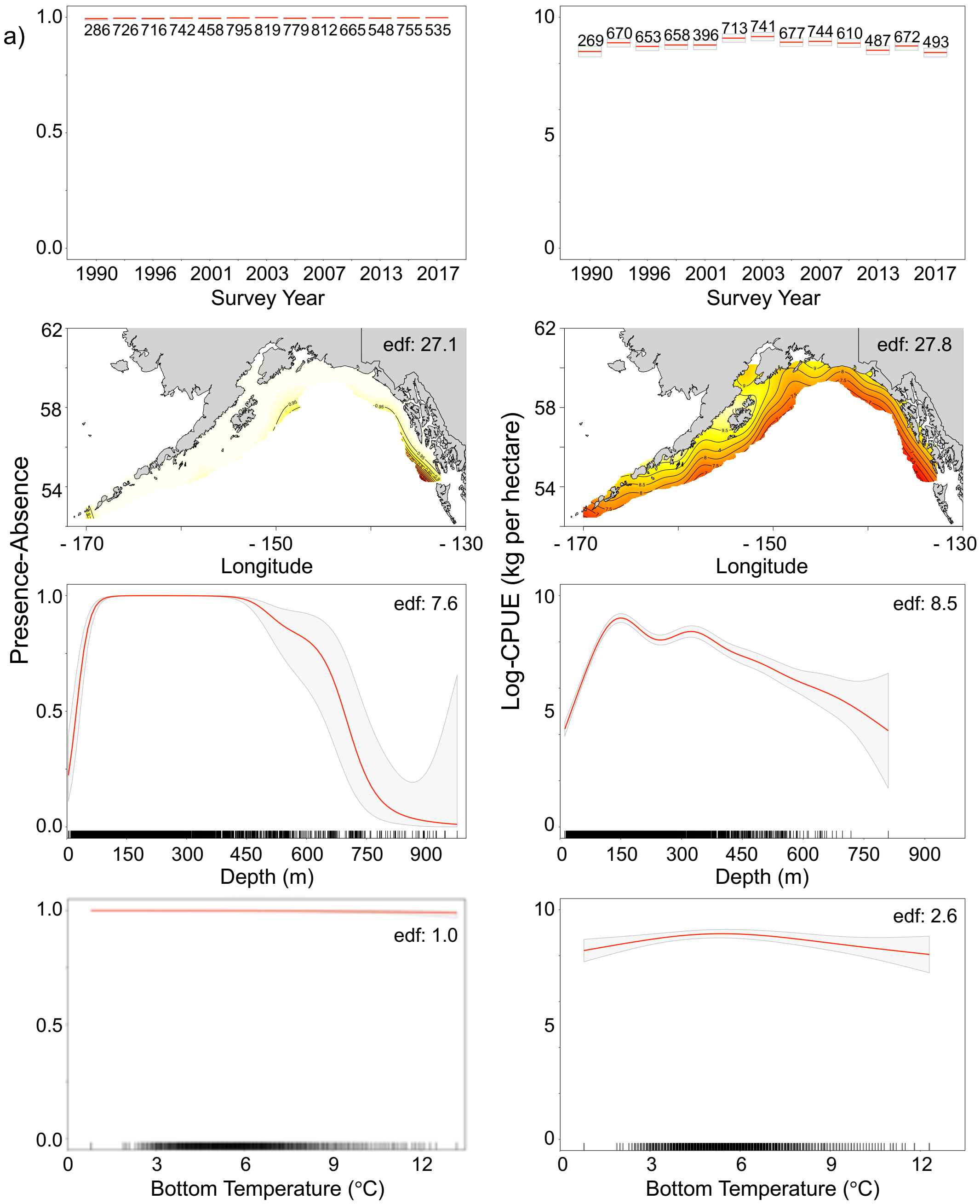

Figure S3. Partial covariate effects on presence-absence (left) and log- CPUE (right) for a) Arrowtooth Flounder, b) Pacific Cod, c) Pacific Halibut, d) Sablefish, and e) Walleye Pollock (Gulf of Alaska , 1990 to 2015). Effective degrees of freedom (edf) are shown for smoothed covariates. Gray bands denote 95% confidence intervals. Sample sizes are noted above or below survey years. Maps: Yellow illustrates greater probability or relative biomass. Red denotes lower probability of occurrence or relative biomass.

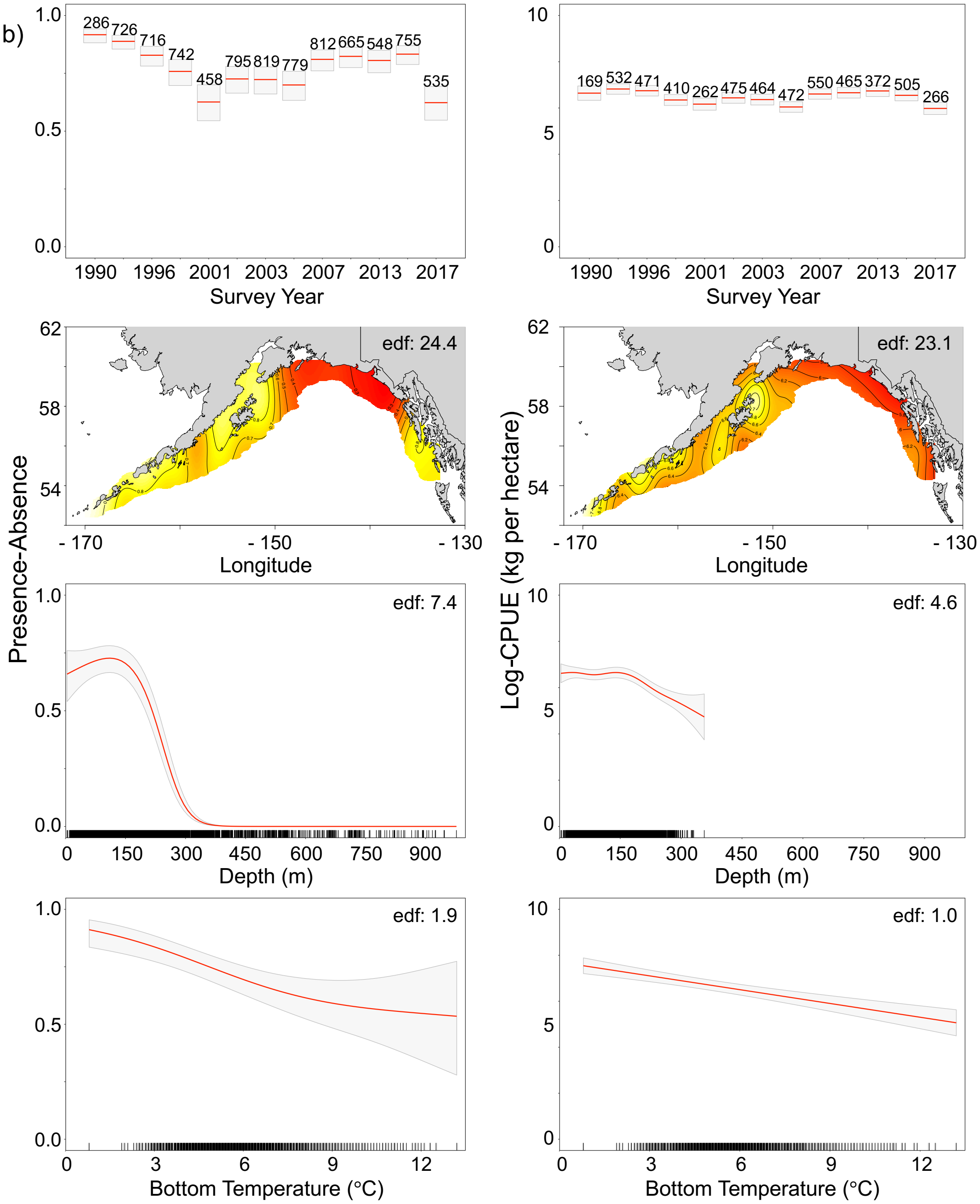

Figure S3 (cont). Partial covariate effects on presence-absence (left) and log- CPUE (right) for a) Arrowtooth Flounder, b) Pacific Cod, c) Pacific Halibut, d) Sablefish, and e) Walleye Pollock (Gulf of Alaska , 1990 to 2015). Effective degrees of freedom (edf) are shown for smoothed covariates. Gray bands denote 95% confidence intervals. Sample sizes are noted above or below survey years. Maps: Yellow illustrates greater probability or relative biomass. Red denotes lower probability of occurrence or relative biomass.

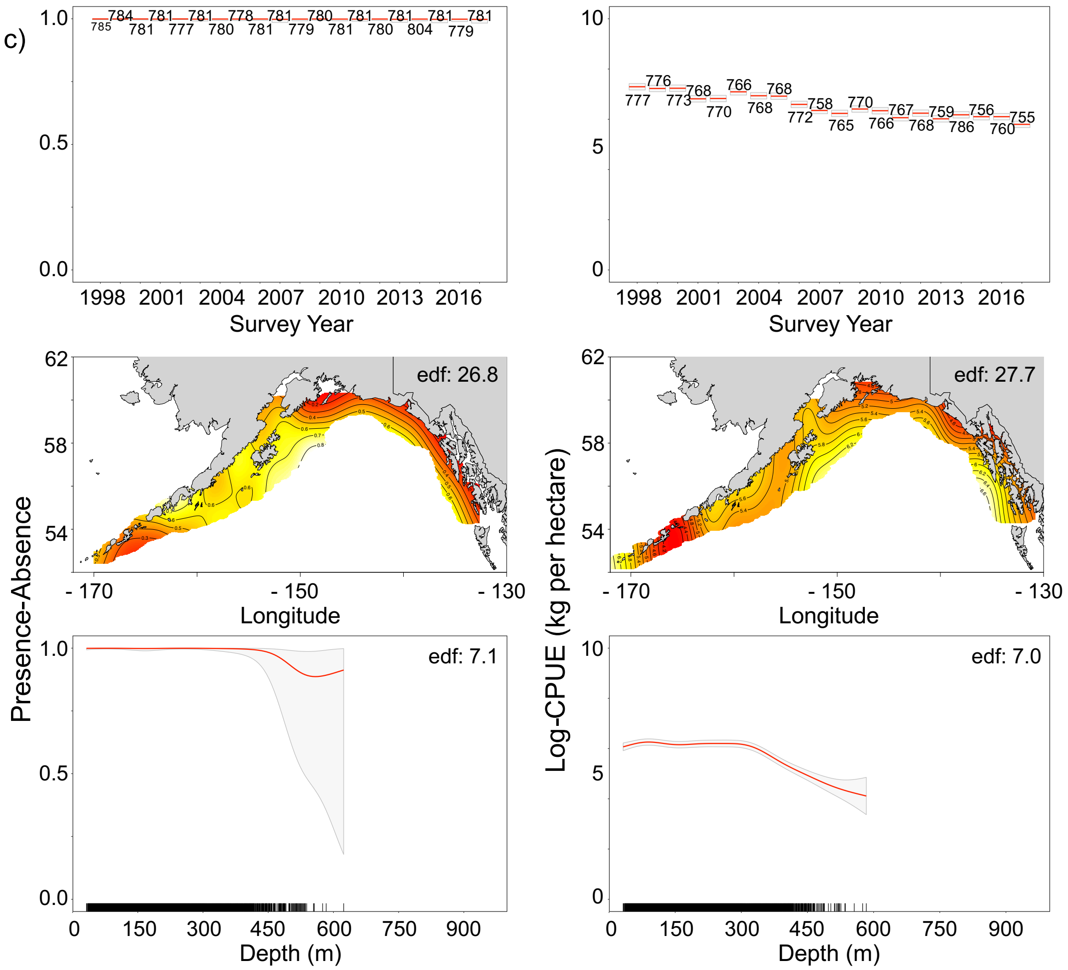

Figure S3 (cont). Partial covariate effects on presence-absence (left) and log- CPUE (right) for a) Arrowtooth Flounder, b) Pacific Cod, c) Pacific Halibut, d) Sablefish, and e) Walleye Pollock (Gulf of Alaska , 1990 to 2015). Effective degrees of freedom (edf) are shown for smoothed covariates. Gray bands denote 95% confidence intervals. Sample sizes are noted above or below survey years. Maps: Yellow illustrates greater probability or relative biomass. Red denotes lower probability of occurrence or relative biomass.

d)

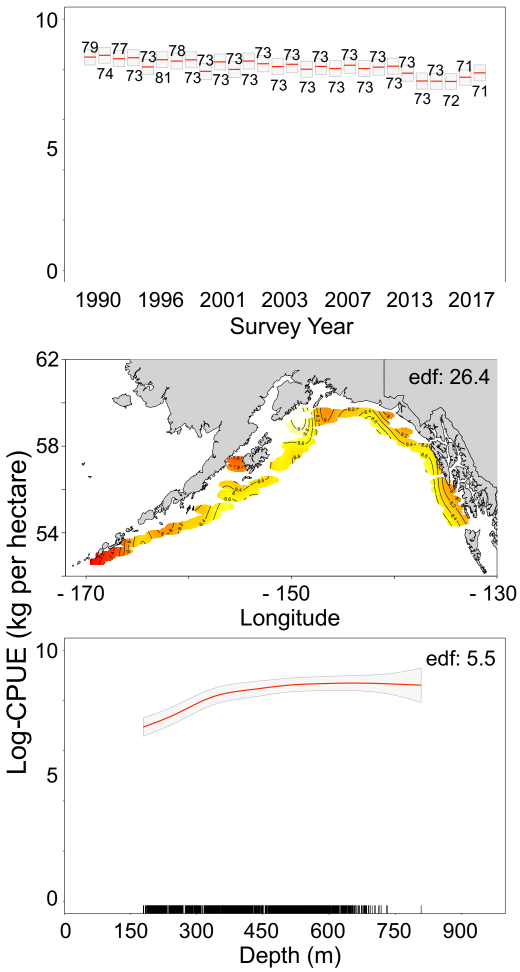

Figure S3 (cont). Partial covariate effects on presence-absence (left) and log- CPUE (right) for a) Arrowtooth Flounder, b) Pacific Cod, c) Pacific Halibut, d) Sablefish, and e) Walleye Pollock (Gulf of Alaska , 1990 to 2015). Effective degrees of freedom (edf) are shown for smoothed covariates. Gray bands denote 95% confidence intervals. Sample sizes are noted above or below survey years. Maps: Yellow illustrates greater probability or relative biomass. Red denotes lower probability of occurrence or relative biomass.

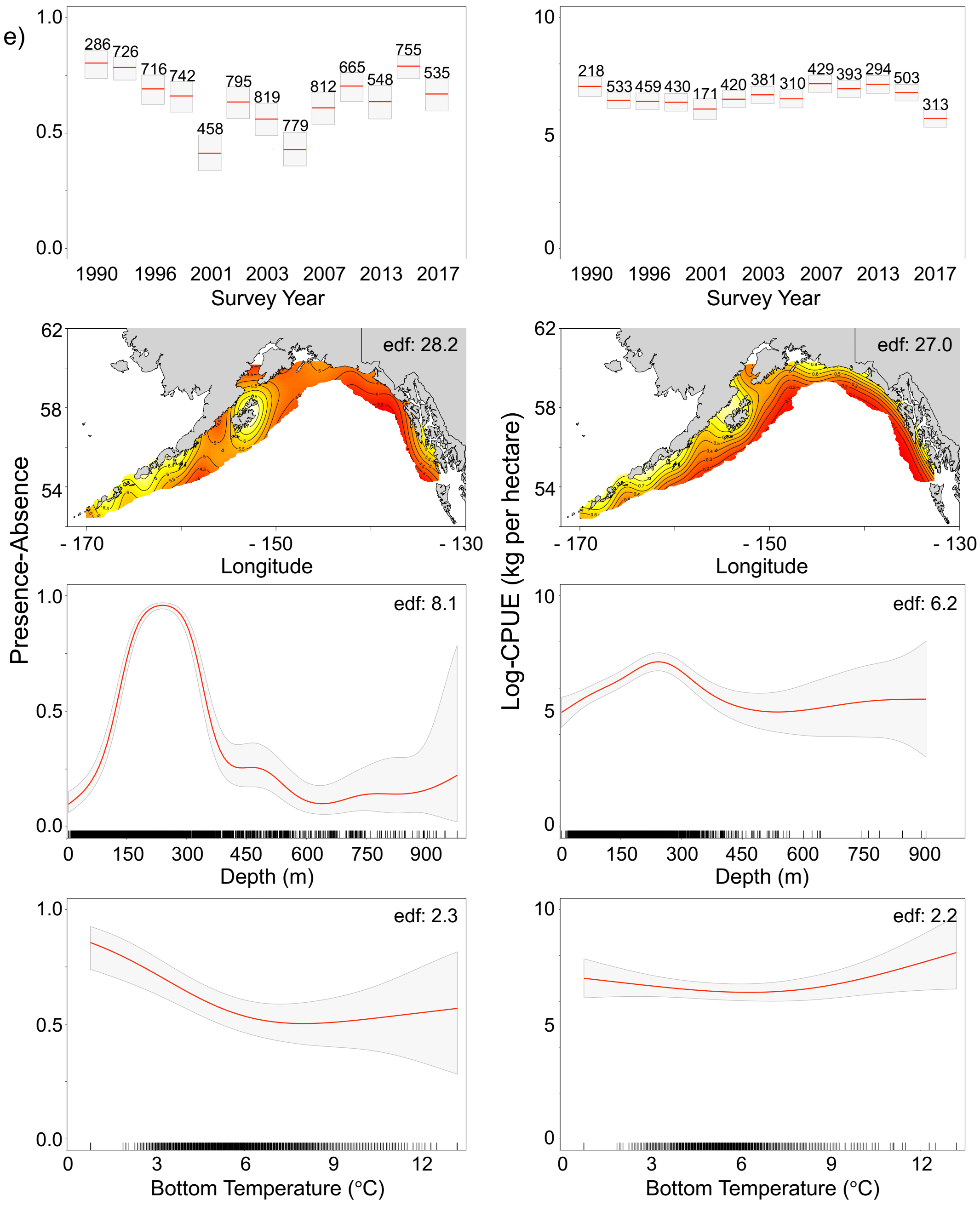

Figure S3 (cont). Partial covariate effects on presence-absence (left) and log- CPUE (right) for a) Arrowtooth Flounder, b) Pacific Cod, c) Pacific Halibut, d) Sablefish, and e) Walleye Pollock (Gulf of Alaska , 1990 to 2015). Effective degrees of freedom (edf) are shown for smoothed covariates. Gray bands denote 95% confidence intervals. Sample sizes are noted above or below survey years. Maps: Yellow illustrates greater probability or relative biomass. Red denotes lower probability of occurrence or relative biomass.

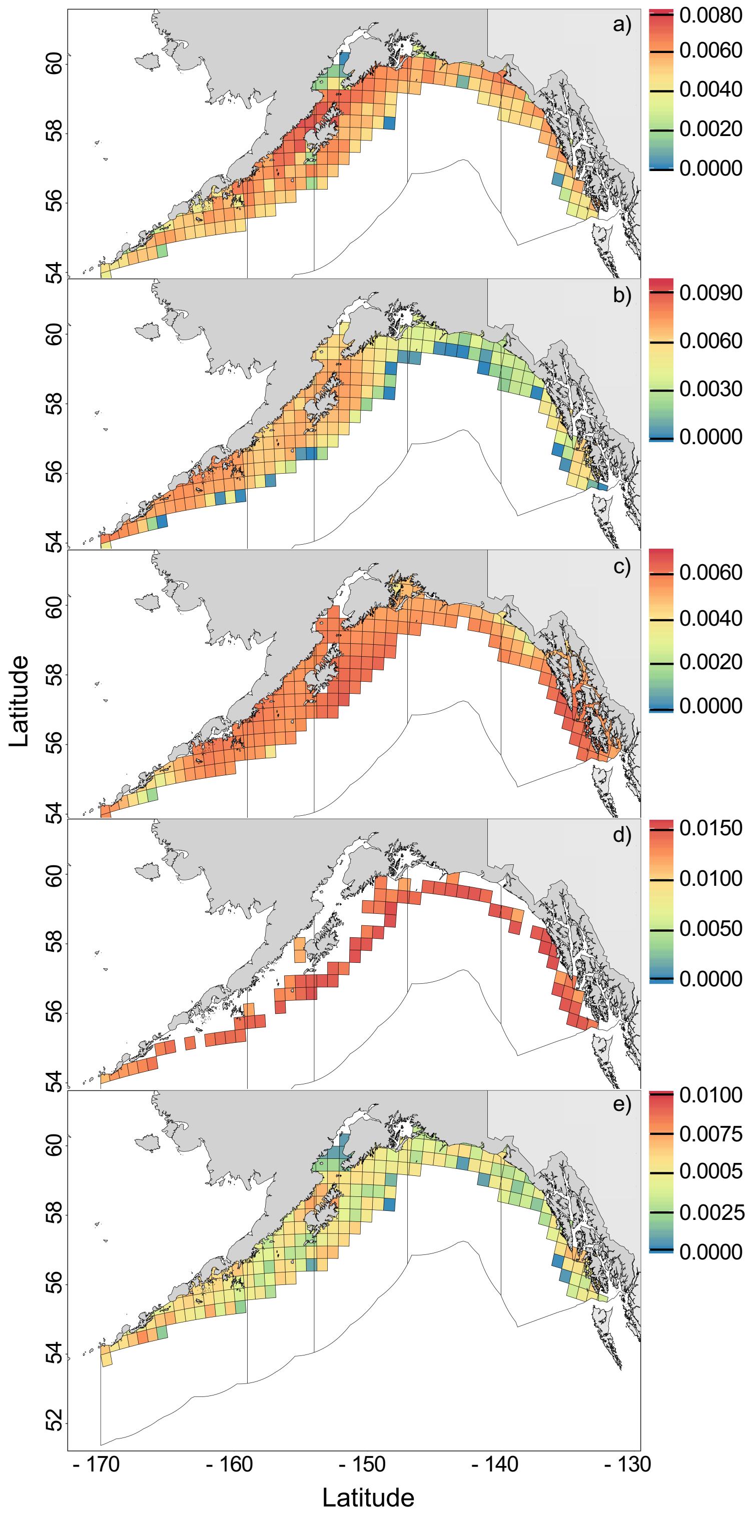

Figure S4. Relative predator densities $rD_{s,i,j}$ for a) Arrowtooth Flounder, b) Pacific Cod, c) Pacific Halibut, d) Sablefish, and e) Walleye Pollock in the Gulf of Alaska (1990 to 2015).

Warm colors indicate higher relative densities and cool colors indicate lower relative densities.

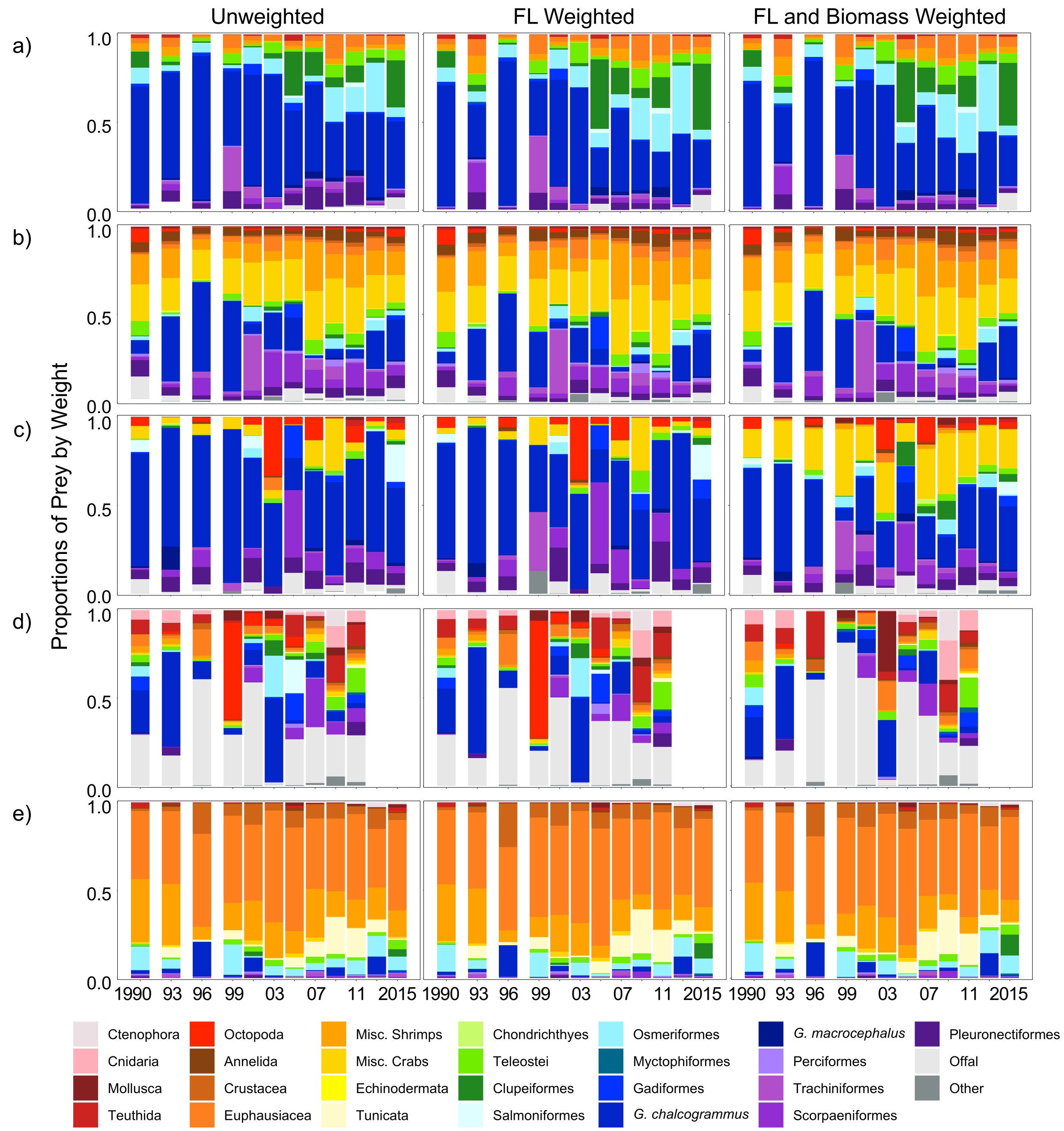

Figure S5. Proportions of prey by weight for a) Arrowtooth Flounder ≥ 19 cm, b) Pacific Cod ≥ 0 cm, c) Pacific Halibut ≥ 82 cm, d) Sablefish ≥ 45 cm, and e) Walleye Pollock ≥ 37 cm (Gulf of Alaska, 1990 to 2015). Diet compositions are shown as unweighted (left panel), fork length-weighted (FL; middle panel), and fork length- and biomass-weighted (right panel). Walleye Pollock prey are shown in dark blue.

LITERATURE CITED

Barbeaux, S., K. Aydin, B. Fissel, K. Holsman, and W. Palsson. 2017. Assessment of the Pacific cod stock in the Gulf of Alaska. North Pacific Fishery Management Council Gulf of Alaska SAFE Report 189–332.

Brodziak, J. 2012. Fitting length-weight relationships with linear regression using the log-transformed allometric model with bias-correction. NOAA Technical Memorandum NMFS-PIFSC H-12-03.

Clark, W. G., and S. R. Hare. 2006. Assessment and management of Pacific halibut: data, methods, and policy. International Pacific Halibut Commission Scientific Report 83.

Dorn, M., K. Aydin, B. Fissel, D. Jones, A. McCarthy, W. Palsson, and K. Spalinger. 2017. Assessment of the Walleye Pollock stock in the Gulf of Alaska. North Pacific Fishery Management Council Gulf of Alaska SAFE Report 47–182.

Hanselman, D. H., C. J. Rodgveller, C. R. Lunsford, and K. H. Fenske. 2017. Assessment of the Sablefish stock in Alaska. North Pacific Fishery Management Council Bering Sea, Aleutian Islands, and Gulf of Alaska SAFE Report 327–502.

Harvey, C. J. 2009. Effects of temperature change on demersal fisheries in the California Current: a bioenergetics approach. Canadian Journal of Fisheries and Aquatic Sciences 66:1449–1461.

Holsman, K. K., and K. Aydin. 2015. Comparative methods for evaluating climate change impacts on the foraging ecology of Alaskan groundfish. Marine Ecology Progress Series 521:217–235.

Holsman, K.K., K. Aydin, J. Sullivan, and T. Hurst, and G. H. Kruse. 2019. Climate effects and bottom-up controls on growth and size-at-age of Pacific halibut (*Hippoglossus stenolepis*). Fisheries Oceanography 28:345–358.

Livingston, P. A., K. Aydin, T. W. Buckley, G. M. Lang, M-S. Yang, and B. S. Miller. 2017. Quantifying food web interactions in the North Pacific – a data-based approach. Environmental Biology of Fishes 100(4):443–470.

Sigler, M. F., and H. H. Zenger Jr. 1989. Assessment of Gulf of Alaska Sablefish and other groundfish based on the domestic longline survey, 1987. NOAA Technical Memorandum NMFS-AFSC Report 169.

Spies, I., K. Aydin, J. N. Ianelli, and W. Palsson. 2017. Assessment of the Arrowtooth Flounder stock in the Gulf of Alaska. North Pacific Fishery Management Council Gulf of Alaska SAFE Report 749–846.

von Szalay, P. G., and N. W. Raring. 2016. Data report: 2015 Gulf of Alaska bottom trawl survey. NOAA Technical Memorandum NMFS-AFSC-325.
